# Substantive model compatible multilevel multiple imputation: A joint modeling approach

**DOI:** 10.1002/sim.9549

**Published:** 2022-08-12

**Authors:** Matteo Quartagno, James R. Carpenter

**Affiliations:** ^1^ Institute for Clinical Trials and Methodology University College London London UK; ^2^ Department of Medical Statistics London School of Hygiene and Tropical Medicine London UK

**Keywords:** joint modeling, missing data, multilevel, multiple imputation

## Abstract

**Background:**

Substantive model compatible multiple imputation (SMC‐MI) is a relatively novel imputation method that is particularly useful when the analyst's model includes interactions, non‐linearities, and/or partially observed random slope variables.

**Methods:**

Here we thoroughly investigate a SMC‐MI strategy based on joint modeling of the covariates of the analysis model. We provide code to apply the proposed strategy and we perform an extensive simulation work to test it in various circumstances.

We explore the impact on the results of various factors, including whether the missing data are at the individual or cluster level, whether there are non‐linearities and whether the imputation model is correctly specified. Finally, we apply the imputation methods to the motivating example data.

**Results:**

SMC‐JM appears to be superior to standard JM imputation, particularly in presence of large variation in random slopes, non‐linearities, and interactions. Results seem to be robust to slight mis‐specification of the imputation model for the covariates. When imputing level 2 data, enough clusters have to be observed in order to obtain unbiased estimates of the level 2 parameters.

**Conclusions:**

SMC‐JM is preferable to standard JM imputation in presence of complexities in the analysis model of interest, such as non‐linearities or random slopes.

## 1 INTRODUCTION

The issues raised by missing data for statistical analyses are well known.^1^ The validity of statistical inference depends on which data are missing (eg, outcome^2^ vs covariates^3^) and for what reasons (eg, completely at random or because of the value of some variables^4^). In the last 30 years, multiple imputation^5^ (MI) has become the standard approach in several fields, including clinical,^6^ and social^7^ research, because it is a computationally practical route to valid and efficient inference under the most general assumption possible with the available data, that is, that data are missing at random (MAR^8^) given what we observed. Furthermore it can be easily extended to explore the sensitivity of results when this assumption is not met.^9^

Unlike other methods, such as inverse probability weighting,^10^ MI allows the analyst to use the same model for the final analysis as they would have used in presence of a fully observed dataset. We call this the *substantive model*. An important condition, though, is that this substantive model has to be compatible^11^ with the model used for imputation in order for the combined procedure of imputation and analysis to yield valid estimates of the substantive model parameters. For example, various studies have shown that a multilevel imputation model has to be used if one aims to analyze the data with a multilevel substantive model.^12^, ^13^

Using a non‐compatible imputation model may therefore introduce additional bias, possibly without eliminating the one due to missing data. There are various situations, though, where compatibility is not easy to achieve, for example when the substantive model includes non‐linearities (eg, a quadratic effect), interactions or a combination of these. Another important example is when data are missing in a variable that is included with a random slope in the substantive analysis model. In such situations, it's been proven that a simple multilevel imputation model may still introduce bias.^14^, ^15^ For these situations, a new type of MI has become increasingly popular in recent years: substantive‐model‐compatible multiple imputation^11^, ^15^, ^16^, ^17^, ^18^ (SMC‐MI). Unlike standard MI, SMC‐MI requires the user to know which substantive model they want to fit on the data in advance of the imputation. At the imputation stage, SMC‐MI implements a theoretically justified probabilistic selection favoring proposed imputation values that are more “compatible” with this model, for example through rejection sampling or a Metropolis‐Hastings algorithm.

The first software developed for SMC‐MI was smcfcs, available for both Stata^19^ and R.^20^ As the name suggests, this uses full conditional specification^21^ to impute the missing data, and it allows for the imputation of single level data only. More recently, few packages have been developed for imputation compatible with multilevel models.^17^, ^22^, ^23^ The aim of this article is twofold: first, we present one of these methods,^24^ which is based on the use of joint modeling imputation^25^ and which is implemented in the R package jomo ^26^ (exploiting the full flexibility of the package, for example, allowing random covariance matrices), providing code to use it in various situations and examples. Second, we evaluate it extensively through a range of challenging simulations based on data from a cluster randomized trial.

## 2 DATA

All the simulations in this article are based on a real data example, with the data coming from a cluster randomized trial whose results were reported in Ayieko et al.^27^ The main goal of the trial was to evaluate the effectiveness of a multifaceted intervention to improve admission pediatric care in Kenyan hospitals. A substantial amount of information was collected to explore secondary scientific questions as well and (not atypically) a considerable proportion of data ended up being missing.

Our analysis focuses on one of the secondary questions of interest, which is whether the use, at admission, of a specially developed structured pediatric admission record (PAR) form could improve the completeness of PARs, the latter measured on a % scale from 0% to 100%, with 100% meaning that all the appropriate pediatric clinical admission information was recorded.

The data is structured as follows: there are admission records for successive children (level 1), nested in the admitting clinician (level 2), and nested in the hospital (level 3). Table 1, taken from Carpenter and Kenward,^5^ Chapter 9, shows the missingness proportions in some of the principal baseline variables collected in the study. These include both level 1, child‐level, variables (PAR form use, child gender and age) and level 2, clinician‐level, variables (gender, years of experience, and attendance in continuing medical education CME).

**TABLE 1:** Descriptive statistics of set of considered variables and relative missingness proportions

	Hospital								
	1	2	3	4	5	6	7	8	% miss.
No. of admissions	1197	1210	1188	1007	798	798	1116	1035	
Completion (mean %)	89	72	88	83	32	49	63	75	0
PAR use (%)	97	82	96	92	22	52	60	97	0
Female child (%)	42	39	41	39	44	38	44	41	6
Female clinician (%)	76	60	52	70	64	92	58	54	14
Clinician's years of experience (median)	0.5	0	0	4	3	3	0	0	20
Clinician's attendance at continuing medical education (%)	46	20	35	59	CME not available in the hospital				9

The main question we wish to explore here is whether use of the structured PAR form improved completeness of admission records, after adjusting for a group of baseline variables: child gender, hospital treatment group, clinician's gender, years of experience, and attendance at CME (continual medical education) training sessions. In particular, we are interested to explore a possible interaction between use of the specially developed PAR form and CME attendance. Since data are clustered in clinicians and hospitals, we use a mixed effects model. In particular, we consider a random‐intercept 3‐level model:

(1) $$y_{\%\text{compl},i,j,h}=\beta_{0}+\sum_{k}\beta_{k}x_{k,i,j,h}+v_{h}+u_{j,h}+w_{i,j,h},$$

where i indexes children (level 1), j clinicians (level 2), h hospitals (level 3), and k the baseline covariates included in the model, that is, PAR use and child gender (level 1), admitting clinician's gender, years of experience, and attendance to CME (level 2), hospital treatment status (level 3) and the cross‐level interaction between CME attendance and PAR use. The two random intercepts at the clinician ($u_{j,h})$ and hospital ($v_{h})$ level are assumed to follow normal distributions with mean 0 and variances $\sigma_{j}$ and $\sigma_{h}$, but the effect of PAR use is the same for all clinicians. Finally, $w_{i,j,h}$ denotes the residual error terms.

We additionally consider a random coefficient model where the use of PAR is allowed to have a varying effect depending on clinician, and where an additional quadratic effect is allowed for clinician's years of experience:

(2) $$y_{\%\text{compl},i,j,h}=\beta_{0}+\sum_{k}\beta_{k}x_{k,i,j,h}+v_{h}+u_{j,h,0}+u_{j,h,1}{\mathrm{PAR}}_{i,j}+w_{i,j,h},$$

where, now, the clinician‐level random coefficients $u_{j,h,0}$ and $u_{j,h,1}$ follow a bivariate normal distribution with unstructured covariance matrix $\Omega_{j}$, as it is the default in the R package for fitting multilevel models lme4,^28^ but not for example in Stata.^29^

Because of the large amount of missing covariate data (particularly at level 2), a complete records analysis omits a substantial proportion of information.^27^ Ideally, we need to use MI consistent with both the multilevel structure and the interaction, in particular correctly handling the random slope on the PAR term.

The rest of the article is organized as follows: in the next section, we present different imputation models that can be used under the joint modeling framework to impute missing multilevel data. We then evaluate these different methods through an extensive simulation study derived from the above cluster randomized trial, providing code in the Supplementary material for users to perform similar analyses. Finally, we provide the results of the two analysis models presented above, when using these new imputation strategies to impute missing data which are compatible with the substantive model.

## 3 METHODS

Consider, for simplicity, a simplified version of models (1) and (2) above, where only clinician‐level random effects are considered, and we only have two level 1 covariates, PAR use and child age (scaled):

(3) $$y_{\%\text{compl},i,j}=\beta_{0}+\beta_{1}{\mathrm{Age}}_{i,j}+\beta_{2}{\mathrm{PAR}}_{i,j}+u_{j}+w_{i,j}$$

(4) $$y_{\%\text{compl},i,j}=\beta_{0}+\beta_{1}{\mathrm{Age}}_{i,j}+\beta_{2}{\mathrm{PAR}}_{i,j}+u_{j,0}+u_{j,1}{\mathrm{PAR}}_{i,j}+w_{i,j.}$$

Assuming both covariates are partially observed, we can consider imputing them with substantive model compatible joint modeling MI.^30^ This uses an explicit formulation of a joint imputation model for the whole data, allowing for (more straightforward^31^) considerations around compatibility of imputation and analysis model in the multilevel setting, at minimal cost in imputation flexibility.

The multivariate normal model is the most simple joint model, and in order to include binary variables (like PAR use) a latent normal variables approach has been developed^32^ and investigated.^33^ A simple joint imputation model is therefore:

(5) $$\left\{\begin{array}{c} y_{\%\text{compl},i,j}=\alpha_{0}+u_{0,j}+\epsilon_{0,i,j}\\ z_{\mathrm{PAR},i,j}=\alpha_{1}+u_{1,j}+\epsilon_{1,i,j}\\ x_{\mathrm{age},i,j}=\alpha_{2}+u_{2,j}+\epsilon_{2,i,j} \end{array}\;\left(\begin{array}{c} \epsilon_{0,i,j}\\ \epsilon_{1,i,j}\\ \epsilon_{2,i,j} \end{array}\right)∼N\left(\left(\begin{array}{c} 0\\ 0\\ 0 \end{array}\right),\Omega_{1}\right)\;\left(\begin{array}{c} u_{0,j}\\ u_{1,j}\\ u_{2,j} \end{array}\right)∼N\left(\left(\begin{array}{c} 0\\ 0\\ 0 \end{array}\right),\Omega_{2}\right),\right.$$

where $z_{\mathrm{PAR},i,j}$ is the latent normal variable for $x_{\mathrm{PAR},i,j}$, such that $z_{\mathrm{PAR},i,j}<0$ if PAR was not used for that observation and $z_{\mathrm{PAR},i,j}\geq 0$ otherwise. Henceforth, we will refer to this model as JM‐Hom, as it uses a joint model under the homoscedasticity assumption, that is, that the variance‐covariance matrix is the same across all level 2 units (eg, for all clinicians).

In order for two models to be compatible, it should be possible to derive one from the other by conditioning on certain variables. If we take (5) and condition on PAR use and age, from simple laws of conditional probabilities of multivariate normal distributions we find:

$$E\left[y_{\%\text{compl},i,j}|\mathrm{PAR},\mathrm{age}\right]=\alpha_{0}+u_{0,j}+\left(\omega_{01}\;\omega_{02}\right)\left(\begin{array}{cc} \omega_{1}^{2} & \omega_{1,2}\\ \omega_{2,1} & \omega_{2}^{2} \end{array}\right)\left(\begin{array}{c} z_{\mathrm{PAR},i,j}-\alpha_{1}+u_{1,j}\\ x_{\mathrm{age},i,j}-\alpha_{2}+u_{2,j} \end{array}\right)$$

Hence, the effect of PAR on the mean of the outcome depends on the variance‐covariance elements only, which are fixed under the homoscedastic model. For this reason, imputation model (5) is compatible with substantive model (3), but not with substantive model (4), because of the random slope, which allows the effect of PAR use to vary between clinicians.

A simple solution is to make the variance‐covariance matrix cluster‐specific, allowing for a cluster‐specific effect of PAR on the outcome in the imputation model. To allow for small clusters, it is possible to further assume that the various variance‐covariance matrices follow a certain distribution, for example the inverse‐Wishart distribution:

(6) $$\Omega_{1,j}∼\mathrm{IW}\left(a,A\right)$$

This was proposed by Yucel,^34^ and investigated further in a series of papers.^35^, ^36^, ^37^ However, though potentially an improvement, particularly for large values of random slope variances, it is still not fully compatible, as the association between variables has different distributions in the two models. Furthermore, it relies on the presence of a moderate (>20) number of decently sized (>50) clusters in order to estimate the variance‐covariance matrices well.^36^ We will refer to this imputation model as JM‐Het, as it assumed heteroscedasticity across clusters.

In order to achieve full compatibility, we need to factor the joint distribution of the outcome and the covariates:

(7) $$f\left(Y,X_{\mathrm{PAR}},X_{\mathrm{AGE}}\right)=f_{y}\left(Y|X_{\mathrm{PAR}},X_{\mathrm{AGE}}\right)f_{x}\left(X_{\mathrm{PAR}},X_{\mathrm{AGE}}\right)$$

This way, it is possible to use the substantive model itself, for example (3) or (4), directly as the conditional distribution of the outcome given the covariates, $f_{y}\left(Y|X_{\mathrm{PAR}},X_{\mathrm{AGE}}\right)$. Not only does this allows for the inclusion of random slopes, but also of polynomial effects, interactions and non‐linear substantive models (eg, Cox proportional hazards), all situations in which finding a compatible joint model is otherwise difficult, if not impossible.Since this method requires the choice of a substantive model in advance of imputation, and later guarantees the compatibility of this model with the imputation model, it is a substantive model compatible imputation strategy, and we henceforth refer to it as SMC‐JM.

### 3.1 Implementation

We have thus far introduced three possible imputation models, but how does the actual imputation work? Standard joint modeling, as in models (5) and (6), works by fitting a MCMC sampler to impute the missing data multiple times, treating missing data as additional parameters and using a data augmentation algorithm.^38^ All parameters, including missing data, are initialized and given a prior, and successively a new value is drawn repeatedly for one set of parameters from the conditional distribution given the current values of all other parameters and the observed data. The sampler is run for several iterations until convergence, and subsequently the currently drawn values for the missing data are used as the first imputation. After another series of iterations, to guarantee stochastic independence, remaining imputations are registered similarly.

When all the conditional distributions used to draw the new parameter values are known, it is possible to use the simplest possible MCMC method, Gibbs sampling. With continuous multilevel data only, for example, a Gibbs sampler using data augmentation has been derived.^39^ In other situations, it is necessary to use an acceptance/rejection step for values drawn from a distribution that is just an approximation to the actual conditional distribution. In these situations, we use a Metropolis‐Hastings sampler. This is the case, for example, when using the latent normal algorithm to include categorical variables; because of constraints to the variance‐covariance matrix elements, the conditional distribution of the matrix given all other parameters is not of a known form, and so it has to be updated using a Metropolis‐Hastings step.^40^

The SMC‐JM model can be fitted with a similar MCMC, but missing data in the covariates have to be imputed with a further Metropolis‐Hastings step, evaluating the effect that the new imputations have on the likelihood of the whole model, which includes the substantive one. Therefore, imputations which increase the likelihood of the substantive model are more likely to be accepted. The algorithm is described in detail in Goldstein et al.^16^ Note that for the imputation of level 2 variables, the effect of the new draw is evaluated on the sum of the log‐likelihood contributions for all the individuals from the same cluster, rather than on a single (log‐)likelihood like in the case of level 1 variables.

Standard and SMC‐JM imputation are both implemented in the R package jomo, that we are going to use for our simulations in the remainder of the article. Both imputation strategies are available in the homoscedastic and heteroscedastic version, in functions jomo and jomo.smc.

## 4 SIMULATIONS

We give an overall ADEMP^41^ summary of the simulation study we undertake, before explaining in more detail the various scenarios investigated.

**Aim:** To evaluate SMC‐JM for the imputation of missing multilevel data and to compare it against the two other multilevel JM imputation methods. In particular, we aim to investigate its behavior in presence of interactions, non‐linearities or random slopes for partially observed covariables in the imputation model, and for varying effect sizes and cluster dimensions.

**Data‐generating mechanism**: We use various data‐generating mechanisms and, in order to use realistic data‐generating parameters, we tune them by fitting two separate models on the complete records of the Ayieko et al^27^ data: a joint model for the covariates, and the substantive model.

Since this data‐generating model corresponds to (7), we additionally check sensitivity of results to different data‐generating mechanisms for the covariates. We do not vary the mechanism for the outcome, as if the substantive model was not correct, this would be a problem for the analysis more than for the imputation itself. The precise parameter values used are listed in Table 2 and in the tables in the Supplementary material.

**TABLE 2:** Results of simulations for random intercept and random slope base‐case scenarios

	$\beta_{0}$					$\beta_{1}$					$\beta_{2}$					$\sigma_{u,00}$	$\sigma_{u,01}$	$\sigma_{u,11}$
Method	Mean	% rel. Bias	Model SE	Emp SE	% Cov	Mean	% rel. Bias	Model SE	Emp SE	% Cov	Mean	% rel. Bias	Model SE	Emp SE	% Cov	Mean	Mean	Mean
Random intercept scenario:																		
True value	0.313					0.497					−0.005					0.010		
Full data	0.312	0.224	0.023	0.023	94.3	0.497	0.034	0.007	0.007	95.6	−0.005	0.828	0.003	0.003	94.0	0.010		
Comp. Rec.	0.306	1.980	0.023	0.023	93.3	0.493	0.796	0.009	0.009	93.9	−0.005	1.077	0.004	0.004	95.3	0.010		
JM‐Hom	0.313	0.197	0.023	0.023	94.5	0.495	0.309	0.007	0.007	95.3	−0.005	2.272	0.003	0.003	95.2	0.010		
JM‐Het	0.317	1.471	0.023	0.023	94.2	0.490	1.301	0.009	0.008	93.5	−0.005	0.584	0.004	0.003	97.9	0.010		
SMC‐JM	0.312	0.223	0.023	0.023	94.4	0.497	0.032	0.007	0.007	95.3	−0.005	0.249	0.003	0.003	94.2	0.010		
Random intercept and slope scenario:																		
True value	0.297					0.511					−0.004					0.015	−0.011	0.021
Full data	0.297	0.174	0.029	0.029	93.2	0.510	0.037	0.033	0.034	92.6	−0.004	1.962	0.002	0.002	96.2	0.015	−0.011	0.021
Comp. Rec.	0.292	1.531	0.029	0.029	93.7	0.507	0.655	0.034	0.034	93.3	−0.004	1.211	0.003	0.003	95.5	0.015	−0.011	0.021
JM‐Hom	0.299	0.801	0.029	0.029	93.4	0.508	0.453	0.034	0.034	93.2	−0.004	7.712	0.003	0.002	97.3	0.015	−0.011	0.021
JM‐Het	0.309	4.095	0.032	0.031	92.9	0.497	2.622	0.036	0.035	92.3	−0.004	1.992	0.004	0.003	98.4	0.017	−0.012	0.022
SMC‐JM	0.297	0.180	0.029	0.029	93.1	0.510	0.035	0.033	0.034	92.5	−0.004	1.763	0.003	0.003	95.3	0.015	−0.011	0.021

*Note*: For fixed effect estimates, we provide mean, relative bias (%), model‐based and empirical SE and coverage percentage. For level‐2 variance components we provide mean only.

We always generate 1000 repetitions, and in each we make some data missing according to a MAR mechanism conditional on the outcome, resulting in approximately 30% incomplete cases. The Supplementary material includes a section describing the data generating mechanism in more detail, including equations for the covariate generating mechanisms, missing data mechanisms, and the outcome generation process description.

**Estimands**: we plan to estimate the fixed‐effect parameters and the random effect variances of the substantive analysis models, that is, either (3) or (4).

**Methods**: We compare the three imputation methods introduced in the previous section, ((5), (6), (7)). We refer to them as JM‐Hom, JM‐Het, and SMC‐JM respectively. JM‐Hom is included as the simplest possible multilevel method, and the one most likely to be currently used by practitioners. JM‐Het because it was recently proposed as an improvement to JM‐Hom and because it is very easily accessible with current software, and SMC‐JM as it is our proposed method. Additionally we fit the model on the complete records, that is, on observations with all the three variables available

**Performance measures**: We compare methods in terms of bias (relative and absolute), empirical and model‐based SEs and confidence interval coverage levels. Ideally, a perfect method should yield minimal bias, similar empirical and model‐based SEs, as close as possible, but not lower, than the ones obtained with the full data, and coverage at the nominal level, for example, 95%.

### 4.1 Base‐case scenario: Random intercept model

First, we start from a very simple example, where the substantive model is (3), that is, a random‐intercept model with clustering by clinician and fixed effects for PAR use and child age. Note the precise simulation parameters used for each example are listed in the Supplementary material.

We use the R package jomo ^26^ in order to impute from each of the three models. While a tutorial on the use of models like (5) and (6) has been published,^42^ we provide in the Supplementary material of this article code to impute from model (7), that is, to perform SMC‐JM MI, and successively fit the substantive model (3) on each imputation and apply Rubin's rules using the mitml ^43^ package. Furthermore, the whole code used for the simulations performed here is available on the GitHub page of the first author (https://github.com/Matteo21Q/Missing‐Data/tree/main/Substantive%20Model%20Compatible%20Multilevel%20Multiple%20Imputation/R%20scripts).

**Research question.** Are all three imputation methods unbiased and equally efficient when the substantive model is a random intercept model?

**Results.** As we discussed above, all the three imputation models considered are compatible with this substantive model. Because of this, it does not come as a surprise that all three methods lead to unbiased estimates and good coverage levels (see Table 2). The only slight exception is that, being an overly general model (6) is slightly conservative in the estimate of the SEs, leading to slight over‐covering, particularly for the estimate of the effect of PAR.

### 4.2 Random‐slope models

We then introduce a random slope for PAR use, allowing each clinician's admission performance to be affected differently by this variable. Recall the complication this brings because of the missing values on PAR. Since the random slope variance in the data is quite small, we additionally explore the sensitivity of results to this assumption, multiplying the random slope variance by a factor of 2 or 5, and the corresponding covariance with the random intercept by a factor of $\sqrt{2}$ or $\sqrt{5}$ accordingly.

The code for imputing from SMC‐JM in this example is very similar to the one for the previous section, and is again available in the Supplementary material.

Note that, while the approach is general, in the current software version (2.7.3) it is only possible to pass random‐effects for a single clustering variable in jomo, and only with an unstructured level 2 variance‐covariance matrix.

**Research question:** Is SMC‐JM really necessary when data are missing in a random slope variable? Is JM‐Het not sufficient?

**Results:** In the base‐case example, JM‐Hom and JM‐Het still work quite well both for the estimation of fixed effects and of the random effect variance. However, the estimates of SEs do not closely match the empirical ones, causing slight over‐covering for the PAR effect estimate.

**FIGURE 1:**
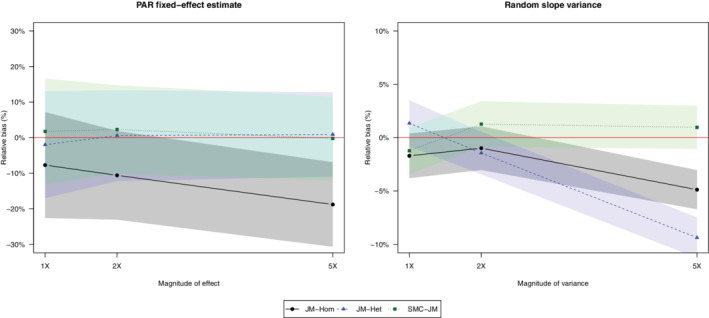
Relative bias (%) in the estimation of (i) fixed effect parameter for PAR use and (ii) random slope variance. Shaded areas indicate the Monte‐Carlo error confidence interval, obtained with formulas from Table 6 of (Morris et al, 2016)

SMC‐JM behaves as expected in these scenarios, and does not seem to be affected by the magnitude of the random effect variance, while JM‐Hom and JM‐Het lead to large bias and low covering proportions for larger random slope variances. Figure 1 shows that, while relative bias is always within Monte‐Carlo error from 0 for SMC‐JM, JM‐Het is only unbiased for the fixed effect parameter estimates while JM‐Hom is biased for both fixed effect estimates and random slope variance. Table (a) in the additional material contains the full results of these simulation scenarios.

### 4.3 Polynomial effects and interactions

We then additionally explore the effect of including a polynomial, for example, quadratic or cubic, or interaction term in the substantive model. The code to perform SMC‐JM in these examples is again quite a straightforward extension of the previous ones, the only difference being in the way we define the analysis model (see Supplementary material).

**Research question:** Can polynomial effects and interactions be straightforwardly handled within the SMC‐JM framework? How much bias do simpler methods introduce?

**Results:** These (Tables (b‐d) in the additional material) are again similar to the previous section, with SMC‐JM being the only method robust to very large polynomial or interaction effects. This is expected, as JM‐Hom and JM‐Het are not built to handle non‐linear relationships.

**FIGURE 2:**
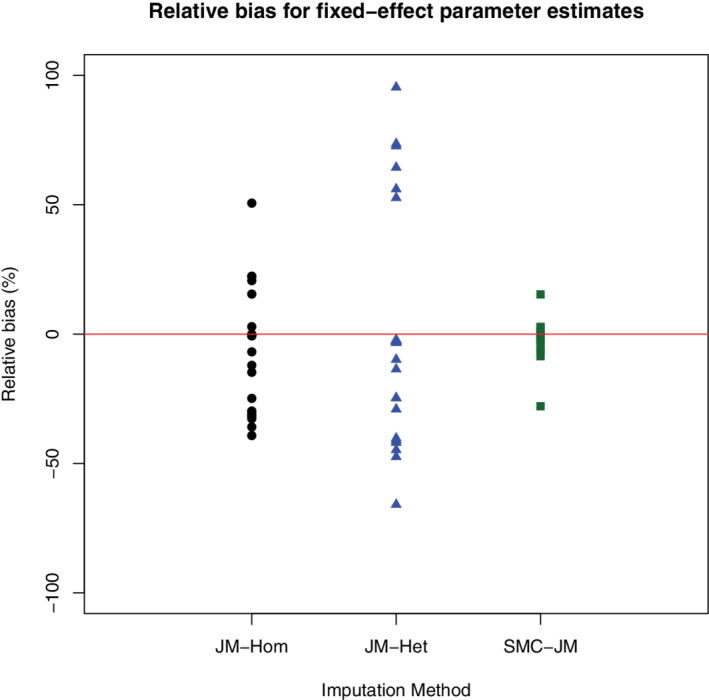
Relative bias (%) in the estimation of all fixed effect parameter estimates for all polynomial and interaction scenarios

Figure 2 helps visualizing these results, by showing the relative bias (%) for estimates of all the fixed effects parameters across all these nine scenarios for the three methods. In particular, the relative bias for all fixed‐effect parameter estimates using SMC‐JM is always within Monte‐Carlo error of 0 (results not shown).

### 4.4 Level 2 variables

Sometimes (as in the motivating example) we might have to impute level‐2 (here clinician level) variables. To explore the behavior of our imputation methods in this scenario, here we include in the imputation model clinician years of experience, instead of child age. In order to impute such variables, it is necessary to tell jomo that they are level 2 variables. This can be done by passing the additional argument level, which indicates whether each variable in the dataset is level 1 or 2 (the level assigned to the clustering indicator variable is irrelevant). Two joint models are then assumed for the covariates, one at level 1 and one at level 2, and the level 2 covariance matrix links the two models by modeling the covariances between level 2 variables and level 1 random effects.

**Research question:** can SMC‐JM work equally well when data are missing at level 2, and hence when the algorithm is slightly modified to account for this?

**FIGURE 3:**
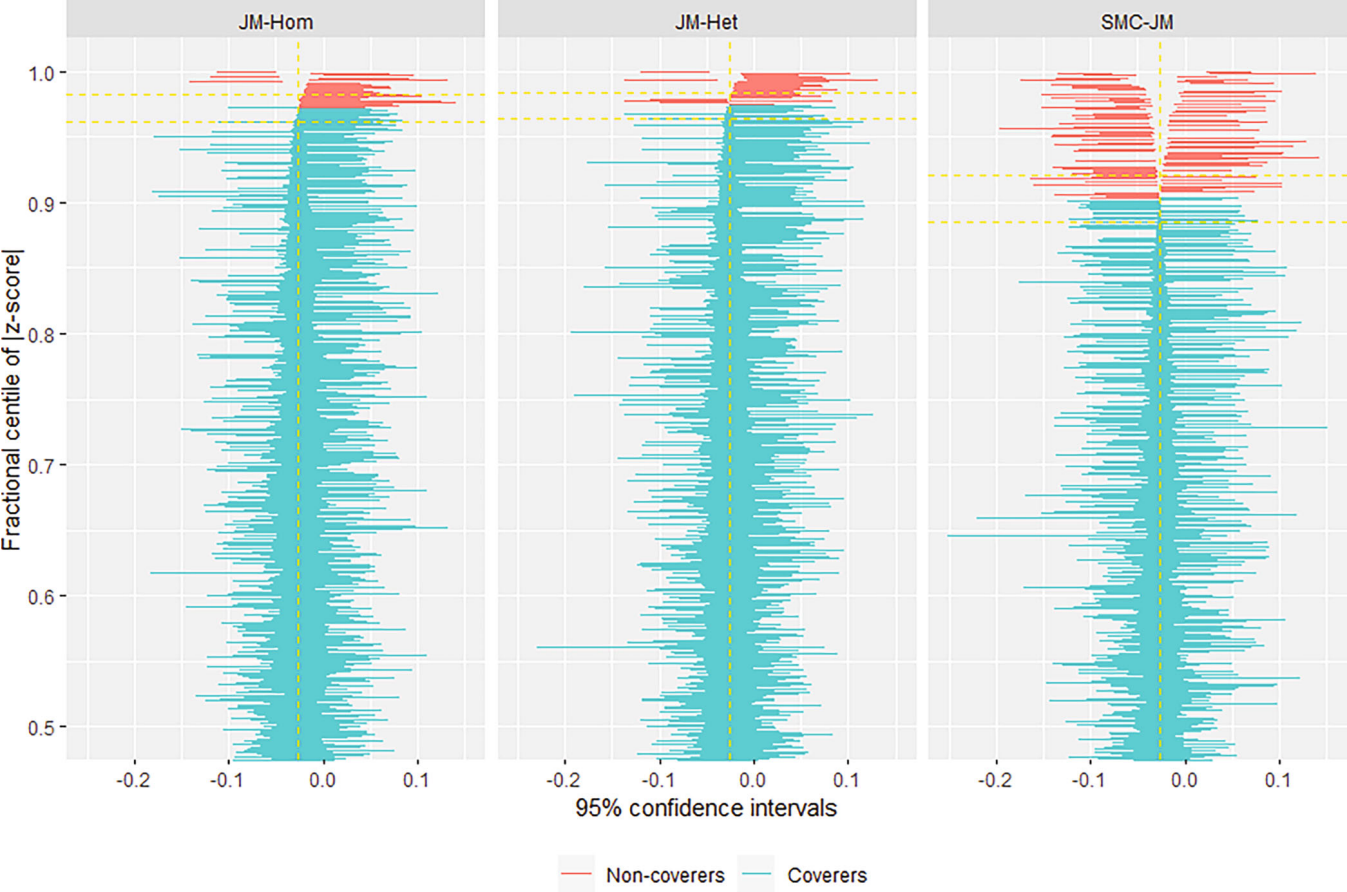
Zip plot of base‐case level‐2 imputation scenario. Blue bars are simulations in which the confidence interval included the true value, which is indicated by the yellow vertical line. Red bars are simulations in which the confidence interval did not include the true value. The yellow horizontal lines indicate the Monte‐Carlo confidence interval for the coverage level

**Results:** These are, at first sight, surprising: JM‐Hom and JM‐Het seem to give better coverage levels than MI.SMC. However, a closer look at the results through Figure 3 (a zipper plot drawn with package rsimsum ^44^), shows that SMC‐JM gives an unbiased estimate of the level 2 variable fixed effect parameter, while the two other methods are biased. The reason for the coverage results is that all three methods are slightly biased in the estimation of the SEs. JM‐Hom and JM‐Het overestimate it, and hence lead to over‐covering despite the bias introduced in the estimate. SMC‐JM, instead, leads to slight underestimation of the error, and hence slight undercovering. When decomposing the MI variance, it appears that the reason for this underestimation is a too small between‐imputation variance.

Interestingly, this problem is alleviated, rather than worsened, when increasing the magnitude of the effect of the level 2 variable on the outcome, so that in the example where the effect of the level 2 covariate on the outcome is 5 times that observed in the complete records, coverage with SMC‐JM is close to that with the full data.

From a closer look at the results, though, it can be noted that the slight bias in the SE estimates, and hence undercovering, is present even with the full data. From further simulations (results not shown) we found that this problem became slightly worse with smaller numbers of clusters and, conversely, gradually disappeared with more clusters. Hence, the likely explanation is that this was due to the well‐known difficulty of fitting multilevel models with few clusters, and to the confounding of the level 2 variable fixed effect with the random effects.

Similar conclusions can be drawn in the example with a cross‐level interaction, and the full results are summarized in Tables (e) and (f) in the additional material.

### 4.5 Auxiliary variables

We then proceed by including auxiliary variables in the data‐generation and imputation models (Table l in the additional material). These are variables that are not in the substantive model but that were collected in the data. They can be included in the imputation model mainly because they can help recovering some information about the missing values; in addition, they may make the MAR assumption more plausible. In this case, we assume the data are MAR even without including the auxiliary variables, but that these are useful predictors of the missing values, and hence can be used to improve precision of the estimates. We generate these variables from two mechanisms, one in which they are associated with PAR use, and one in which they are associated with the outcome.

In order to include auxiliary variables with jomo.lmer, it is enough to include them in the data but not in the substantive model. Note that, if the auxiliary variable is not included in the substantive model this means there is an assumption of conditional independence with the outcome, given the values of the covariates in the substantive model. It is not clear what the implication of this assumption not holding would be on the results, and whether including the auxiliary variables in the outcome model would be preferable. This might be the topic of future research.

**Research question:** Can auxiliary variables be easily incorporated in SMC‐JM and is the effect of their inclusion similar to that in the case of JM‐Hom and JM‐Het?

**Results**: These remain good with SMC‐JM, and the information recovery from auxiliary variables is such that SEs are very close to the ones from full data analysis. JM‐Hom and JM‐Het fail instead in the first example. This is because the mechanism used for the generation of the auxiliary variables is not compatible with the joint models (5) and (6). In particular, this is a general location model with a quite extreme choice of parameters, which has been shown to be problematic (though perhaps not very realistic) for the simple latent normal imputation model.^33^

### 4.6 Substantive models

There are currently three multilevel models supported for SMC‐JM imputation in jomo. In addition to the linear mixed models, illustrated thus far, the package allows for imputation compatible with a logistic mixed effect model (glmer) in case of binary outcome and a cumulative link mixed model (clmm) in case of ordinal outcome.

We generate data from these models, using PAR as outcome for the binary example (substituted by child gender as a covariate) and categorizing completion as a 4‐level ordinal outcome using quartiles to split the continuous variable.

**Research question:** Can we use SMC‐JM with diverse forms of substantive models, rather than just with a linear link and Gaussian error?

**Results**: From Table (i) in additional material, results are good with both models, indicating that conclusions valid for linear mixed model compatible imputation could be extended to other substantive models.

### 4.7 Different data generating mechanisms

In all the scenarios explored thus far, data were generated from a model that could be considered a sub‐type of (7). Generating the data under the correct assumptions for our novel method can obviously lead to better results than with a generic data generating mechanism. Therefore, here we explore the sensitivity of these results to slightly different data generating mechanisms for the covariates. In particular, first we generate PAR from a simple binomial distribution, and scaled age from a normal distribution with mean conditional on PAR value (assuming clinician use of PAR differs by child age). Second, we investigate an example where age is simulated first, and PAR is generated assuming a quadratic relationship with age on the log‐odds scale.

Note that this second example is theoretically not consistent with the imputation model, where a multivariate normal model is used to model PAR and age jointly.

**Research question:** Are results sensitive to the distributional form of the joint imputation model for the covariates of the substantive model?

**Results:** From Table (m) in the additional material, results suggest that there is no practical impact on the conclusions, and that SMC‐JM still works well even under different data generating mechanisms. This is because we are focusing on the estimates of the substantive model only, for which we are imputing compatibly. We cannot use the imputed data to investigate the association between the covariates, though, as this would be biased.

### 4.8 Sensitivity analyses

Finally, here we perform sensitivity analyses to explore how results change with different simulation parameters. Audigier et al^36^ evaluated JM‐Het alongside other imputation strategies^45^, ^46^ based on full conditional specification and found that some factors impacted the quality of results substantially, most importantly number and dimension of clusters, and proportion of systematically missing data. We therefore repeat similar analyses, first investigating the effect of the cluster dimension on results.

**Research questio**n: Is SMC‐JM less sensitive than JM‐Het to small number and dimension of clusters?

**FIGURE 4:**
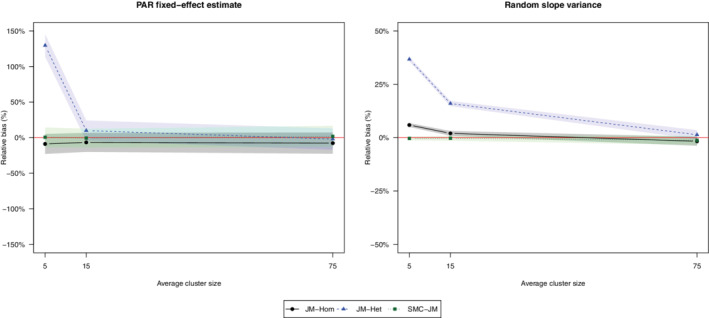
Sensitivity of results to average cluster size. Left panel shows relative bias in estimation of PAR fixed effect estimate, right panel relative bias in estimation of random slope variance

**Results**: As can be seen in Figure 4, while JM‐Het suffers from serious bias for smaller clusters, SMC‐JM seems to be unaffected by the dimension of clusters. This is in line with what was found in Huque et al,^47^ where SMC‐JM was shown to work well even with longitudinal data, a typical situation where cluster size is generally small.

Similarly, the number of clusters seems not to have a huge effect, after considering that, with as few as 6 clusters, even the full data analysis tends to be biased.

Finally, with 20% systematically missing data, that is, data missing for the whole cluster for 20% of clinicians, results with SMC‐JM are very good, while the SEs appear to be slightly overestimated with the other two methods.

## 5 EXAMPLE

We are now ready to apply the three different imputation strategies to handle missing data in the original data set from the Kenyan randomized controlled trial. We already defined the two possible substantive models: a random intercept model (1) and a random coefficient model with a random slope for PAR (2).

Both models include an interaction, variables at both child and clinician‐level, and a third level as well (hospital). Our software does not allow for more than two levels, but since there are only eight hospitals, whose characteristics mean they are not a‐priori exchangeable, we include hospital as a fixed effect in all imputation models.

In order to make comparisons more realistic, rather than just comparing the simple formulations of JM‐Hom and JM‐Het to SMC‐JM, we follow Chapter 9 of Carpenter and Kenward.^5^ For both JM‐Hom and JM‐Het, first of all we impute separately by treatment group and, second, by the interaction groups defined by PAR use, since it is fully observed in the original data and included with an interaction in both substantive models. We therefore have four imputation models for both JM‐Hom and JM‐Het. We impute from each of these four, and we finally combine the imputations in a single dataset before fitting the substantive model and applying Rubin's rules.

For SMC‐JM, instead, we simply impute the whole dataset compatibly with the substantive models (1) and (2).

Table 3 shows the results when fitting the substantive models with the complete records or with data imputed using each of the three models. The results do not differ markedly between the imputation models, but the complete records analysis gives very different conclusions with regards to CME (continuing medical education) attendance. Specifically, while the complete records estimates suggest there is not much difference in completeness of admission forms between doctors who attended or did not attend CME sessions, and that PAR use did not interact with it, the MI estimates suggest attendance to CME alone might even lower completeness, but once clinicians who attended CME use the PAR, completeness increases substantially, suggesting the existence of an interaction. However, while both the main CME effect and its interaction with PAR are strongly significant in model (1), they are not significant using model (2).

**TABLE 3:** Results of substantive model analysis applied to Kenyan cluster randomized trial data

Random‐Interc. model (1)	Complete records			JM‐Hom			JM‐Het			SMC‐JM		
Fixed‐effect par	Est.	SE	*P*‐value	Est.	SE	*P*‐value	Est.	SE	*P*‐value	Est.	SE	*P*‐value
Intercept	26.319	2.302	<0.001	26.247	2.460	<0.001	26.121	2.515	<0.001	26.625	2.300	<0.001
PAR use	50.200	0.429	<0.001	48.746	0.410	<0.001	48.752	0.415	<0.001	48.714	0.407	<0.001
Female child	0.257	0.235	0.274	0.165	0.253	0.516	−0.026	0.314	0.935	0.193	0.232	0.405
Female clinician	0.154	0.878	0.861	0.602	0.995	0.547	0.726	1.023	0.48	0.496	0.880	0.573
Years of experience	−0.330	0.083	<0.001	−0.413	0.203	0.064	−0.378	0.214	0.104	−0.451	0.088	<0.001
Interventional hospital	9.993	3.102	0.016	11.163	3.245	0.001	11.150	3.322	0.001	11.007	3.150	<0.001
CME attendance	0.714	1.713	0.677	−5.089	1.579	0.001	−5.074	1.637	0.002	−5.224	1.602	0.001
PAR x CME interaction	−0.455	1.374	0.740	5.868	1.167	<0.001	5.914	1.165	<0.001	5.958	1.159	<0.001
Var. components:	Est.			Est.			Est.			Est.		
Hospital	17.097			19.031			19.934			17.800		
Clinician	41.059			50.234			50.979			48.002		
Child	88.953			100.153			100.128			100.355		
Random slope model (2)												
Fixed‐effect par	Est.	SE	*P*‐value	Est.	SE	*P*‐value	Est.	SE	*P*‐value	Est.	SE	*P*‐value
Intercept	25.532	2.572	<0.001	25.518	2.677	<0.001	25.249	2.719	<0.001	26.372	2.603	<0.001
PAR use	50.033	1.231	<0.001	48.884	1.098	<0.001	48.912	1.096	<0.001	48.871	1.100	<0.001
Female child	0.193	0.216	0.370	0.176	0.219	0.422	−0.021	0.299	0.945	0.154	0.214	0.471
Female clinician	0.309	1.049	0.769	0.524	1.087	0.63	0.697	1.045	0.505	0.149	1.100	0.892
Years of experience	−0.140	0.305	0.648	−0.393	0.274	0.158	−0.238	0.24	0.323	−0.552	0.297	0.07
Years of exp squared	−0.011	0.013	0.388	−0.002	0.012	0.864	−0.009	0.011	0.409	0.003	0.013	0.814
Interventional hospital	10.565	3.184	0.014	11.196	3.448	0.001	11.219	3.56	0.002	10.909	3.294	0.001
CME attendance	0.679	2.749	0.805	−3.059	2.349	0.193	−3.052	2.357	0.195	−3.224	2.397	0.179
PAR x CME interaction	−0.521	2.842	0.855	4.012	2.509	0.11	4.089	2.502	0.102	4.057	2.531	0.109
Var. components:	Est.			Est.			Est.			Est.		
Hospital	17.116			21.113			22.619			19.014		
Clinician (Int)	136.871			116.188			115.728			115.047		
Clinician (slope)	186.671			185.252			185.222			185.353		
Clinician (corr)	−125.560			−107.110			−106.510			−107.200		
Child	73.658			82.246			82.242			82.246		

*Note*: Results are provided for two substantive models, one with a random‐intercept only and one with a random slope and a quadratic effect for years of experience.

Finally, the only practical difference between imputation methods is that the estimate for the parameter related to years of experience is slightly larger when using SMC‐JM compared to other imputation strategies, and there is now strong evidence that it is different from zero.

## 6 DISCUSSION

In this article we have introduced and used simulations to comprehensively evaluate a two‐level substantive‐model‐compatible multiple imputation algorithm based on joint modeling imputation. Simulation results suggest this can be an improvement over standard MI methods for handling missing multilevel data, particularly in presence of large random slope variances (where the slope is partially observed), polynomial effects and/or interactions in the analysis model of substantive interest.

We have additionally showed that SMC‐JM seems to be less affected by cluster size and number, compared to the JM‐Het method. This is expected, as JM‐Het struggles to estimate cluster‐specific variance parameters once the cluster size becomes very small, and the parameters for the distribution of variances when the cluster number is limited, while SMC‐JM is not affected by this.

We have shown how SMC‐JM leads to unbiased parameter estimates even when imputing level‐2 variables, although some small underestimation of SEs led to under‐covering of the parameter estimates for level 2 variables with a moderately small number of clusters.

The main disadvantage of SMC‐JM compared to standard imputation is that the user needs to know the precise functional form of the substantive model in advance of the imputation. This, first, means that in principle there should be a different imputation for each analysis to be performed, losing one of the advantages of MI over other methods. Second, it makes model selection problematic, for example if the analyst plans to use fractional polynomials. A natural way around this is to perform model selection on a dataset imputed compatibly with the most general possible model, and to possibly repeat imputation compatibly with the selected model at the end. Future work might compare different approaches.

The imputation model presented here assumes a multivariate normal model for all covariates of the substantive model. When more complex associations exist between the covariates, this could theoretically introduce bias. Our sensitivity analysis shows that this is likely not an issue in practice, as long as the substantive model is correctly specified. Grund et al^18^ obtained similar results, as they found that practically meaningful bias only occurs with very strong missingness mechanisms and non‐linear associations between covariates. In the same paper, they also showed that it is important to fully accommodate the substantive model in order for SMC‐JM to lead to unbiased estimates. The current implementation of jomo does not allow, for example, for the inclusion of level‐2 summaries of level‐1 variables in the imputation model, and in such situations including only level 1 variables could introduce some bias.

While jomo currently supports imputation compatible with linear, logistic, and ordinal mixed models, and with Cox proportional hazards models, the methodology could potentially be extended to allow for imputation compatible with any analysis model. We aim in the future to include further functions, for example to impute compatibly with frailty models.

As with all MCMC imputation methods, it is always important to make sure the sampler has converged before starting to register imputations. It is possible to check the convergence of the sampler with the MCMCchain functions in jomo, similarly to what we previously described for the standard JM‐Hom and JM‐Het functions.^42^ With SMC‐JM, focusing on the convergence of the parameters of the substantive model might be enough in practice.

As with most imputation methods, SMC‐JM assumes data are missing at random given the observed data. The method could be extended to allow for sensitivity analyses to different, MNAR, missing data mechanisms.

Multilevel SMC‐MI is now implemented in several packages besides jomo. In particular, the R package mdmb ^23^ implements an algorithm based on sequential imputation, the standalone software Blimp ^17^ uses a slightly different version of JM imputation and the R package JointAI ^22^ uses joint modeling within the Bayesian framework, that is, performing imputation and analysis in the same step. What jomo adds to these packages is the ability to fit heteroscedastic models, which may prove useful particularly in certain settings, for example, individual patient data meta‐analyses.

While our work focused on JM imputation, the idea of SMC‐MI remains the same whatever the imputation framework, that is, whether the user defines the joint distribution for the covariates of the substantive model (as we did), or a set of sequential conditional distributions (as in mdmb) or a set of univariate fully conditional distributions (as in FCS). Hence, when coded to impute compatibly with the same substantive model, all these approaches should lead to similar conclusions.

### 6.1 Conclusions

Substantive model compatible imputation algorithms have all the advantages of standard MI without suffering from compatibility issues. They should become the new standard for imputation whenever the precise functional form of the substantive model is known in advance. The R package jomo provides an implementation of multilevel SMC‐MI based on joint modeling imputation, which consistently performs better than existing JM imputation strategies in presence of interactions, non‐linearity or random slopes in the substantive analysis model.

## FUNDING INFORMATION

This work was supported by the Medical Research Council, with Grant numbers (MC_UU_12023/29) and (MC_UU_00004/07).

## CONFLICT OF INTEREST

The authors declare that there is no conflict of interest

## Supporting information


**Appendix S1** Supporting information

## Data Availability

The code used for the simulations will be made available on the GitHub page of the first author (Matteo21Q).
