# Supplementary material for: Substantive model compatible multilevel multiple imputation: A joint modeling approach
Source: Stat Med. 2022 Aug 12;41(25):5000–15. doi: 10.1002/sim.9549 (PMC9804749; doi:10.1002/sim.9549)
Supplement: Supplementary file 1 — Appendix S1 Supporting information [file SIM-41-5000-s001.docx]

**Data Generating Mechanisms of Simulation scenarios:**

Here we provide the data-generating mechanisms for the covariates for all simulation scenarios. For most scenarios, covariates were obtained by fitting a bivariate joint model with scaled child age and PAR use as outcomes, using latent normal variables to handle the binary PAR use variable. The general form of the model fitted and later used to generate the covariate data is:

$$\left( i \right) \left\{ \begin{matrix} z_{PAR,i,j} =\alpha_{1}+u_{1,j}+\epsilon_{1,i} \\ x_{age,i,j} =\alpha_{2}+u_{2,j}+\epsilon_{2,i} \end{matrix} \left( \begin{matrix} \epsilon_{1,i} \\ \epsilon_{2,i} \end{matrix} \right)\sim N\left( \left( \begin{matrix} 0 \\ 0 \end{matrix} \right),\Omega_{1} \right) \left( \begin{matrix} u_{1,j} \\ u_{2,j} \end{matrix} \right)\sim N\left( \left( \begin{matrix} 0 \\ 0 \end{matrix} \right),\Omega_{2} \right) \right.$$

Fitting this model on the complete records with an MCMC sampler (burn-in=1000 iterations), we found the following parameter values: $\alpha_{1}=$-0.751$, \alpha_{2}=$0.016,$\Omega_{1}=\left( \begin{matrix} 1 & 0.113 \\ 0.113 & 0.987 \end{matrix} \right)$ and $\Omega_{2}=\left( \begin{matrix} 0.768 & -0.0004 \\ -0.0004 & 0.030 \end{matrix} \right)$.

These values were used to generate the covariate for most scenarios. Exceptions were:

- The GLMER and CLMM scenarios, where PAR was used as outcome (GLMER) or was leading to convergence issues for the outcome model. In these examples, a model similar to (i) was fitted but with child gender replacing PAR use. Parameters of this model were: : $\alpha_{1}=$-0.112$, \alpha_{2}=$0.013,$\Omega_{1}=\left( \begin{matrix} 1 & -0.004 \\ -0.004 & 0.986 \end{matrix} \right)$ and $\Omega_{2}=\left( \begin{matrix} 0.033 & -0.003 \\ -0.003 & 0.031 \end{matrix} \right)$.
- The first scenario of sensitivity to different data-generating mechanisms, where PAR use was drawn from a Bernoulli distribution with p=0.3, and scaled age from a normal distribution with mean 0.2*PAR and standard deviation 0.5.
- The second scenario of sensitivity to different data-generating mechanisms, where age was drawn from a normal distribution with mean 0.2 and variance 0.5 and PAR use from a binomial distribution with $p={logit}^{-1}(-0.3+0.6*AGE+0.2*{AGE}^{2})$;
- The level 2 imputation scenarios, where we substituted child age with clinician’s years of experience. For these, we found the following parameter values: $\alpha_{1}=-0.738, \alpha_{2}=$-0.120, ,$\Omega_{1}=1$ and $\Omega_{2}=\left( \begin{matrix} 0.769 & 0.096 \\ 0.096 & 0.933 \end{matrix} \right)$.

In the two auxiliary variables examples, these were generated from distributions conditional on a covariate and the outcome respectively. In particular, for the first example the auxiliary variable was drawn from a normal distribution with mean the child scaled age and standard deviation 0.2. In the second example, it was generated from a normal distribution with the outcome as mean and standard deviation 0.

Finally, the data generating model for the outcome was simply obtained by fitting the substantive model for each scenario on the complete records, and using the estimated parameters to generate the random effects, first, and the outcome later, conditional on covariates and random effects. Record completion proportion was used as outcome when a linear mixed model was the substantive model, PAR use with binomial GLMER while for CLMM, in absence of an ordinal variable, we generated it by committing a statistical sin, splitting continuous record completion proportion in 4 categories, using as splitting points 50%,80% and 90%. Estimated values of all parameters that have been used to generate all outcome data are listed in the table of results for each scenario.

Finally, for all scenarios, except where otherwise stated, we generate 1000 datasets, each formed by 1500 individuals divided in 20 clusters, where each individual can be assigned to either cluster with the same probability (5%). We introduce missing data from a MAR mechanism where the probability of missingness in both PAR and age is $p_{MISS}={(1+e^{2-1.5Y})}^{-1}$, and observations with missing data are drawn from a binomial distribution. This mechanism leads to ~70% complete records after discarding observations with either PAR or age missing.

Table a: Results of random slope scenarios, with sensitivity to larger slope variance than in the base-case scenario.

| Method | $\beta_{0}$ | | | | | $\beta_{1}$ | | | | | $\beta_{2}$ | | | | | $\sigma_{u,00}$ | $\sigma_{u,01}$ | $\sigma_{u,11}$ |
| --- | --- | --- | --- | --- | --- | --- | --- | --- | --- | --- | --- | --- | --- | --- | --- | --- | --- | --- |
|  | Mean | % rel. bias | Model SE | Emp SE | % Cov | Mean | % rel. bias | Model SE | Emp SE | % Cov | Mean | % rel. bias | Model SE | Emp SE | % Cov | Mean | Mean | Mean |
| Random slope scenario, doubled random slope variance: | | | | | | | | |  |  |  |  |  |  |  |  |  |  |
| True value | 0.297 |  |  |  |  | 0.511 |  |  |  |  | -0.004 |  |  |  |  | 0.015 | -0.016 | 0.043 |
| Full Data | 0.296 | 0.205 | 0.029 | 0.028 | 95.1 | 0.512 | 0.203 | 0.047 | 0.045 | 94.7 | -0.004 | 2.250 | 0.002 | 0.002 | 95.4 | 0.015 | -0.016 | 0.043 |
| Comp. Rec. | 0.291 | 1.983 | 0.029 | 0.029 | 95.1 | 0.509 | 0.377 | 0.047 | 0.045 | 94.9 | -0.004 | 1.380 | 0.003 | 0.003 | 94.2 | 0.015 | -0.016 | 0.043 |
| JM-Hom | 0.298 | 0.388 | 0.029 | 0.028 | 95.5 | 0.510 | 0.175 | 0.047 | 0.044 | 95.3 | -0.004 | 10.597 | 0.003 | 0.002 | 97.3 | 0.015 | -0.015 | 0.042 |
| JM-Het | 0.311 | 5.027 | 0.033 | 0.036 | 92.6 | 0.494 | 3.261 | 0.048 | 0.050 | 93.8 | -0.004 | 0.579 | 0.004 | 0.003 | 97.7 | 0.017 | -0.016 | 0.042 |
| SMC-JM | 0.296 | 0.247 | 0.029 | 0.028 | 94.7 | 0.512 | 0.223 | 0.047 | 0.045 | 94.9 | -0.004 | 2.295 | 0.003 | 0.003 | 93.5 | 0.015 | -0.016 | 0.043 |
| Random slope scenario, random slope variance 5 times larger: | | | | | | | | |  |  |  |  |  |  |  |  |  |  |
| True value | 0.297 |  |  |  |  | 0.511 |  |  |  |  | -0.004 |  |  |  |  | 0.015 | -0.025 | 0.106 |
| Full Data | 0.295 | 0.387 | 0.029 | 0.030 | 94.2 | 0.515 | 0.868 | 0.073 | 0.077 | 93.2 | -0.004 | 0.026 | 0.002 | 0.002 | 95.4 | 0.015 | -0.025 | 0.107 |
| Comp. Rec. | 0.290 | 2.077 | 0.029 | 0.030 | 93.2 | 0.511 | 0.159 | 0.073 | 0.076 | 92.9 | -0.004 | 1.036 | 0.003 | 0.003 | 94.9 | 0.015 | -0.025 | 0.105 |
| JM-Hom | 0.300 | 1.080 | 0.030 | 0.031 | 94.2 | 0.511 | 0.050 | 0.072 | 0.077 | 92.6 | -0.003 | 18.802 | 0.003 | 0.002 | 96.9 | 0.016 | -0.023 | 0.101 |
| JM-Het | 0.321 | 8.152 | 0.036 | 0.050 | 90.2 | 0.486 | 4.759 | 0.072 | 0.091 | 89.1 | -0.004 | 0.891 | 0.005 | 0.005 | 98.1 | 0.019 | -0.023 | 0.097 |
| SMC-JM | 0.296 | 0.351 | 0.029 | 0.030 | 94.2 | 0.515 | 0.846 | 0.073 | 0.077 | 93.2 | -0.004 | 0.232 | 0.003 | 0.003 | 94.3 | 0.016 | -0.025 | 0.108 |

Table b: Results of quadratic effect scenarios, with sensitivity to larger effect than in the base-case scenario. We provide estimates for slope parameters and level 2 variance components.

| Method | $\beta_{1}$ | | | | | $\beta_{2}$ | | | | | $\beta_{3}$ | | | | | $\sigma_{u,00}$ | $\sigma_{u,01}$ | $\sigma_{u,11}$ |
| --- | --- | --- | --- | --- | --- | --- | --- | --- | --- | --- | --- | --- | --- | --- | --- | --- | --- | --- |
|  | Mean | % rel. bias | Model SE | Emp SE | % Cov | Mean | % rel. bias | Model SE | Emp SE | % Cov | Mean | % rel. bias | Model SE | Emp SE | % Cov | Mean | Mean | Mean |
| Quadratic effect scenario: | | | | | | | | |  |  |  |  |  |  |  |  |  |  |
| True value | 0.510 |  |  |  |  | -0.001 |  |  |  |  | -0.002 |  |  |  |  | 0.015 | -0.011 | 0.021 |
| Full Data | 0.509 | 0.253 | 0.034 | 0.034 | 93.9 | -0.001 | 9.410 | 0.002 | 0.002 | 94.9 | -0.002 | 1.946 | 0.002 | 0.002 | 94.5 | 0.015 | -0.011 | 0.021 |
| Comp. Rec. | 0.506 | 0.843 | 0.034 | 0.035 | 93.3 | -0.001 | 3.287 | 0.003 | 0.003 | 94.8 | -0.002 | 6.718 | 0.002 | 0.002 | 94.1 | 0.015 | -0.011 | 0.021 |
| JM-Hom | 0.507 | 0.699 | 0.034 | 0.034 | 94.4 | -0.001 | 15.463 | 0.003 | 0.003 | 97.7 | -0.001 | 39.211 | 0.002 | 0.001 | 98.6 | 0.016 | -0.011 | 0.021 |
| JM-Het | 0.496 | 2.900 | 0.036 | 0.036 | 92.3 | -0.001 | 64.375 | 0.004 | 0.003 | 98.0 | -0.001 | 65.852 | 0.002 | 0.001 | 97.9 | 0.017 | -0.012 | 0.022 |
| SMC-JM | 0.509 | 0.241 | 0.034 | 0.034 | 93.7 | -0.001 | 6.274 | 0.003 | 0.003 | 94.8 | -0.001 | 8.558 | 0.002 | 0.002 | 93.5 | 0.015 | -0.011 | 0.021 |
| Quadratic effect twice as large: | | | | | | | | |  |  |  |  |  |  |  |  |  |  |
| True value | 0.510 |  |  |  |  | -0.001 |  |  |  |  | -0.003 |  |  |  |  | 0.015 | -0.011 | 0.021 |
| Full Data | 0.510 | 0.147 | 0.034 | 0.034 | 93.9 | -0.001 | 6.024 | 0.002 | 0.002 | 95.7 | -0.003 | 2.801 | 0.002 | 0.002 | 95.5 | 0.015 | -0.011 | 0.021 |
| Comp. Rec. | 0.507 | 0.711 | 0.034 | 0.034 | 93.9 | -0.001 | 3.383 | 0.003 | 0.003 | 94.8 | -0.003 | 2.026 | 0.002 | 0.002 | 95.3 | 0.015 | -0.011 | 0.021 |
| JM-Hom | 0.507 | 0.605 | 0.034 | 0.033 | 94.6 | -0.001 | 22.262 | 0.003 | 0.003 | 96.9 | -0.002 | 31.593 | 0.002 | 0.001 | 97.8 | 0.016 | -0.011 | 0.021 |
| JM-Het | 0.496 | 2.794 | 0.036 | 0.035 | 93.3 | -0.002 | 73.544 | 0.004 | 0.003 | 98.4 | -0.002 | 47.426 | 0.002 | 0.001 | 96.1 | 0.017 | -0.012 | 0.022 |
| SMC-JM | 0.510 | 0.152 | 0.034 | 0.034 | 94.1 | -0.001 | 2.003 | 0.003 | 0.003 | 94.6 | -0.003 | 1.152 | 0.002 | 0.002 | 95.4 | 0.015 | -0.011 | 0.021 |
| Quadratic effect 5 times as large: | | | | | |  |  |  |  |  |  |  |  |  |  |  |  |  |
| True value | 0.510 |  |  |  |  | -0.001 |  |  |  |  | -0.008 |  |  |  |  | 0.015 | -0.011 | 0.021 |
| Full Data | 0.509 | 0.192 | 0.034 | 0.034 | 92.9 | -0.001 | 22.614 | 0.002 | 0.002 | 94.9 | -0.008 | 0.108 | 0.002 | 0.002 | 95.4 | 0.015 | -0.011 | 0.022 |
| Comp. Rec. | 0.506 | 0.837 | 0.034 | 0.034 | 93.2 | -0.001 | 26.481 | 0.003 | 0.003 | 94.9 | -0.008 | 1.133 | 0.002 | 0.002 | 95.0 | 0.015 | -0.011 | 0.021 |
| JM-Hom | 0.507 | 0.653 | 0.034 | 0.034 | 93.3 | -0.001 | 2.877 | 0.003 | 0.003 | 97.2 | -0.005 | 32.675 | 0.002 | 0.001 | 79.7 | 0.016 | -0.011 | 0.022 |
| JM-Het | 0.496 | 2.824 | 0.036 | 0.036 | 92.8 | -0.002 | 72.652 | 0.004 | 0.003 | 97.5 | -0.005 | 41.970 | 0.002 | 0.002 | 74.7 | 0.017 | -0.012 | 0.022 |
| SMC-JM | 0.509 | 0.191 | 0.034 | 0.034 | 92.9 | -0.001 | 27.891 | 0.003 | 0.003 | 95.0 | -0.008 | 0.943 | 0.002 | 0.002 | 94.9 | 0.015 | -0.011 | 0.022 |

Table c: Results of cubic effect scenarios, with sensitivity to larger effect than in the base-case scenario. We provide estimates for slope parameters and level 2 variance components.

| Method | $\beta_{1}$ | | | | | $\beta_{2}$ | | | | | $\beta_{3}$ | | | | | $\sigma_{u,00}$ | $\sigma_{u,01}$ | $\sigma_{u,11}$ |
| --- | --- | --- | --- | --- | --- | --- | --- | --- | --- | --- | --- | --- | --- | --- | --- | --- | --- | --- |
|  | Mean | % rel. bias | Model SE | Emp SE | % Cov | Mean | % rel. bias | Model SE | Emp SE | % Cov | Mean | % rel. bias | Model SE | Emp SE | % Cov | Mean | Mean | Mean |
| Cubic effect scenario: | | | | | | | | |  |  |  |  |  |  |  |  |  |  |
| True value | 0.510 |  |  |  |  | -0.002 |  |  |  |  | 0.000 |  |  |  |  | 0.015 | -0.011 | 0.021 |
| Full Data | 0.511 | 0.176 | 0.034 | 0.034 | 94.0 | -0.002 | 7.680 | 0.004 | 0.004 | 95.8 | 0.000 | 3.146 | 0.001 | 0.001 | 93.3 | 0.015 | -0.011 | 0.021 |
| Comp. Rec. | 0.508 | 0.399 | 0.034 | 0.034 | 92.8 | -0.002 | 15.486 | 0.005 | 0.005 | 95.0 | 0.000 | 9.544 | 0.001 | 0.001 | 94.6 | 0.015 | -0.011 | 0.021 |
| JM-Hom | 0.509 | 0.263 | 0.034 | 0.034 | 94.2 | -0.002 | 22.377 | 0.004 | 0.004 | 98.3 | 0.000 | 30.042 | 0.001 | 0.001 | 98.2 | 0.016 | -0.011 | 0.021 |
| JM-Het | 0.497 | 2.556 | 0.036 | 0.035 | 93.1 | -0.003 | 56.042 | 0.005 | 0.004 | 98.9 | 0.000 | 44.757 | 0.001 | 0.001 | 99.7 | 0.017 | -0.012 | 0.022 |
| SMC-JM | 0.511 | 0.164 | 0.034 | 0.034 | 93.9 | -0.002 | 15.317 | 0.005 | 0.005 | 93.8 | 0.000 | 5.420 | 0.001 | 0.001 | 93.5 | 0.016 | -0.011 | 0.021 |
| Cubic effect twice as large: | | | | | | | | |  |  |  |  |  |  |  |  |  |  |
| True value | 0.510 |  |  |  |  | -0.002 |  |  |  |  | -0.001 |  |  |  |  | 0.015 | -0.011 | 0.021 |
| Full Data | 0.512 | 0.232 | 0.033 | 0.034 | 93.3 | -0.002 | 3.835 | 0.004 | 0.004 | 94.8 | -0.001 | 1.703 | 0.001 | 0.001 | 95.3 | 0.015 | -0.011 | 0.021 |
| Comp. Rec. | 0.509 | 0.303 | 0.034 | 0.035 | 93.0 | -0.002 | 8.131 | 0.005 | 0.005 | 96.6 | -0.001 | 9.300 | 0.001 | 0.001 | 95.2 | 0.015 | -0.011 | 0.021 |
| JM-Hom | 0.510 | 0.180 | 0.034 | 0.034 | 94.4 | -0.002 | 20.659 | 0.004 | 0.003 | 98.8 | 0.000 | 31.798 | 0.001 | 0.001 | 98.8 | 0.015 | -0.011 | 0.021 |
| JM-Het | 0.499 | 2.318 | 0.036 | 0.035 | 93.8 | -0.003 | 52.656 | 0.005 | 0.004 | 98.9 | 0.000 | 41.082 | 0.001 | 0.001 | 99.3 | 0.017 | -0.012 | 0.022 |
| SMC-JM | 0.512 | 0.237 | 0.034 | 0.034 | 93.2 | -0.002 | 0.633 | 0.005 | 0.004 | 95.3 | -0.001 | 2.591 | 0.001 | 0.001 | 94.4 | 0.015 | -0.011 | 0.021 |
| Cubic effect 5 times as large: | | | | | |  |  |  |  |  |  |  |  |  |  |  |  |  |
| True value | 0.510 |  |  |  |  | -0.002 |  |  |  |  | -0.002 |  |  |  |  | 0.015 | -0.011 | 0.021 |
| Full Data | 0.510 | 0.084 | 0.034 | 0.033 | 94.7 | -0.002 | 2.872 | 0.004 | 0.004 | 95.4 | -0.002 | 0.243 | 0.001 | 0.001 | 95.0 | 0.015 | -0.011 | 0.022 |
| Comp. Rec. | 0.507 | 0.656 | 0.034 | 0.033 | 94.6 | -0.002 | 6.100 | 0.005 | 0.005 | 95.6 | -0.002 | 0.812 | 0.001 | 0.001 | 95.6 | 0.015 | -0.011 | 0.021 |
| JM-Hom | 0.508 | 0.494 | 0.034 | 0.032 | 94.9 | -0.003 | 50.602 | 0.005 | 0.003 | 98.3 | -0.001 | 30.917 | 0.001 | 0.001 | 98.8 | 0.016 | -0.011 | 0.022 |
| JM-Het | 0.497 | 2.578 | 0.036 | 0.034 | 94.0 | -0.003 | 95.352 | 0.005 | 0.004 | 98.5 | -0.001 | 40.258 | 0.001 | 0.001 | 97.5 | 0.017 | -0.012 | 0.022 |
| SMC-JM | 0.510 | 0.095 | 0.034 | 0.033 | 94.8 | -0.002 | 0.205 | 0.005 | 0.004 | 95.6 | -0.002 | 0.800 | 0.001 | 0.001 | 94.9 | 0.015 | -0.011 | 0.022 |

Table d: Results of interaction scenarios, with sensitivity to larger effect than in the base-case scenario. We provide estimates for slope parameters and level 2 variance components.

| Method | $\beta_{1}$ | | | | | $\beta_{2}$ | | | | | $\beta_{3}$ | | | | | $\sigma_{u,00}$ | $\sigma_{u,01}$ | $\sigma_{u,11}$ |
| --- | --- | --- | --- | --- | --- | --- | --- | --- | --- | --- | --- | --- | --- | --- | --- | --- | --- | --- |
|  | Mean | % rel. bias | Model SE | Emp SE | % Cov | Mean | % rel. bias | Model SE | Emp SE | % Cov | Mean | % rel. bias | Model SE | Emp SE | % Cov | Mean | Mean | Mean |
| Interaction scenario: | | | | | | | | |  |  |  |  |  |  |  |  |  |  |
| True value | 0.509 |  |  |  |  | -0.010 |  |  |  |  | 0.007 |  |  |  |  | 0.015 | -0.011 | 0.021 |
| Full Data | 0.509 | 0.133 | 0.033 | 0.035 | 92.8 | -0.010 | 2.215 | 0.005 | 0.005 | 96.1 | 0.007 | 1.690 | 0.005 | 0.005 | 95.8 | 0.015 | -0.011 | 0.021 |
| Comp. Rec. | 0.506 | 0.660 | 0.034 | 0.036 | 92.9 | -0.010 | 3.289 | 0.006 | 0.006 | 94.7 | 0.007 | 3.393 | 0.007 | 0.007 | 93.9 | 0.015 | -0.011 | 0.021 |
| JM-Hom | 0.507 | 0.528 | 0.034 | 0.035 | 93.3 | -0.009 | 6.859 | 0.005 | 0.005 | 96.6 | 0.006 | 12.064 | 0.006 | 0.005 | 97.9 | 0.015 | -0.011 | 0.021 |
| JM-Het | 0.495 | 2.761 | 0.036 | 0.036 | 92.9 | -0.009 | 3.361 | 0.008 | 0.006 | 98.6 | 0.006 | 13.544 | 0.008 | 0.006 | 98.8 | 0.017 | -0.012 | 0.022 |
| SMC-JM | 0.509 | 0.128 | 0.034 | 0.035 | 93.0 | -0.010 | 2.824 | 0.005 | 0.005 | 94.6 | 0.007 | 2.403 | 0.006 | 0.006 | 94.7 | 0.015 | -0.011 | 0.021 |
| Interaction effect twice as large: | | | | | | | | |  |  |  |  |  |  |  |  |  |  |
| True value | 0.509 |  |  |  |  | -0.010 |  |  |  |  | 0.014 |  |  |  |  | 0.015 | -0.011 | 0.021 |
| Full Data | 0.511 | 0.313 | 0.034 | 0.034 | 93.2 | -0.009 | 1.470 | 0.005 | 0.004 | 95.5 | 0.014 | 2.053 | 0.005 | 0.005 | 95.6 | 0.015 | -0.011 | 0.022 |
| Comp. Rec. | 0.508 | 0.240 | 0.034 | 0.035 | 93.0 | -0.009 | 1.543 | 0.006 | 0.006 | 94.5 | 0.014 | 2.292 | 0.007 | 0.007 | 94.5 | 0.015 | -0.011 | 0.021 |
| JM-Hom | 0.509 | 0.067 | 0.034 | 0.034 | 93.8 | -0.008 | 14.755 | 0.005 | 0.005 | 97.0 | 0.010 | 24.836 | 0.006 | 0.005 | 95.5 | 0.015 | -0.011 | 0.021 |
| JM-Het | 0.498 | 2.221 | 0.036 | 0.035 | 93.6 | -0.009 | 9.830 | 0.008 | 0.006 | 98.7 | 0.010 | 24.654 | 0.008 | 0.006 | 97.7 | 0.017 | -0.012 | 0.022 |
| SMC-JM | 0.511 | 0.331 | 0.034 | 0.034 | 93.3 | -0.009 | 1.130 | 0.005 | 0.005 | 94.1 | 0.014 | 2.361 | 0.006 | 0.006 | 94.6 | 0.015 | -0.011 | 0.022 |
| Interaction effect 5 times as large: | | | | | |  |  |  |  |  |  |  |  |  |  |  |  |  |
| True value | 0.509 |  |  |  |  | -0.010 |  |  |  |  | 0.035 |  |  |  |  | 0.015 | -0.011 | 0.021 |
| Full Data | 0.510 | 0.087 | 0.033 | 0.036 | 92.2 | -0.010 | 0.719 | 0.005 | 0.005 | 94.3 | 0.035 | 0.470 | 0.005 | 0.006 | 94.3 | 0.015 | -0.011 | 0.021 |
| Comp. Rec. | 0.507 | 0.502 | 0.034 | 0.037 | 91.7 | -0.010 | 0.774 | 0.006 | 0.006 | 94.1 | 0.034 | 0.443 | 0.007 | 0.007 | 95.0 | 0.015 | -0.011 | 0.021 |
| JM-Hom | 0.507 | 0.392 | 0.034 | 0.036 | 93.0 | -0.006 | 35.858 | 0.006 | 0.005 | 93.0 | 0.024 | 29.726 | 0.006 | 0.005 | 66.2 | 0.016 | -0.011 | 0.021 |
| JM-Het | 0.497 | 2.486 | 0.036 | 0.038 | 90.8 | -0.007 | 24.687 | 0.008 | 0.006 | 97.4 | 0.025 | 29.011 | 0.008 | 0.006 | 80.5 | 0.017 | -0.012 | 0.021 |
| SMC-JM | 0.510 | 0.083 | 0.034 | 0.036 | 92.4 | -0.010 | 0.210 | 0.005 | 0.005 | 94.4 | 0.035 | 0.068 | 0.006 | 0.006 | 94.3 | 0.015 | -0.011 | 0.021 |

Table e: Results of level 2 data scenarios, with sensitivity to larger effect than in the base-case scenario. We provide estimates for all parameters and level 2 variance components.

| Method | $\beta_{0}$ | | | | | $\beta_{1}$ | | | | | $\beta_{2}$ | | | | | $\sigma_{u,00}$ | $\sigma_{u,01}$ | $\sigma_{u,11}$ |
| --- | --- | --- | --- | --- | --- | --- | --- | --- | --- | --- | --- | --- | --- | --- | --- | --- | --- | --- |
|  | Mean | % rel. bias | Model SE | Emp SE | % Cov | Mean | % rel. bias | Model SE | Emp SE | % Cov | Mean | % rel. bias | Model SE | Emp SE | % Cov | Mean | Mean | Mean |
| Level 2 imputation scenario: | | | | | | | | |  |  |  |  |  |  |  |  |  |  |
| True value | 0.301 |  |  |  |  | 0.519 |  |  |  |  | -0.026 |  |  |  |  | 0.016 | -0.012 | 0.020 |
| Full Data | 0.300 | 0.376 | 0.029 | 0.030 | 93.9 | 0.521 | 0.382 | 0.032 | 0.032 | 95.0 | -0.025 | 3.740 | 0.021 | 0.023 | 93.2 | 0.016 | -0.012 | 0.020 |
| Comp. Rec. | 0.298 | 0.843 | 0.035 | 0.034 | 93.5 | 0.519 | 0.001 | 0.038 | 0.037 | 94.5 | -0.025 | 3.893 | 0.026 | 0.029 | 91.0 | 0.015 | -0.012 | 0.020 |
| JM-Hom | 0.301 | 0.226 | 0.031 | 0.030 | 95.6 | 0.519 | 0.010 | 0.033 | 0.032 | 95.0 | -0.019 | 25.084 | 0.026 | 0.021 | 97.2 | 0.016 | -0.012 | 0.020 |
| JM-Het | 0.311 | 3.575 | 0.033 | 0.033 | 94.7 | 0.508 | 2.111 | 0.035 | 0.035 | 94.8 | -0.020 | 23.321 | 0.027 | 0.022 | 97.4 | 0.017 | -0.013 | 0.020 |
| SMC-JM | 0.300 | 0.274 | 0.030 | 0.030 | 93.1 | 0.521 | 0.363 | 0.032 | 0.032 | 95.0 | -0.025 | 3.748 | 0.025 | 0.028 | 90.3 | 0.015 | -0.012 | 0.020 |
| Level 2 variable effect twice as large: | | | | | | | | |  |  |  |  |  |  |  |  |  |  |
| True value | 0.301 |  |  |  |  | 0.519 |  |  |  |  | -0.052 |  |  |  |  | 0.016 | -0.012 | 0.020 |
| Full Data | 0.300 | 0.206 | 0.029 | 0.029 | 95.4 | 0.520 | 0.165 | 0.033 | 0.032 | 94.1 | -0.051 | 2.606 | 0.022 | 0.025 | 90.2 | 0.015 | -0.012 | 0.020 |
| Comp. Rec. | 0.298 | 1.065 | 0.035 | 0.035 | 94.3 | 0.519 | 0.009 | 0.039 | 0.04 | 92.5 | -0.051 | 2.719 | 0.026 | 0.031 | 90.2 | 0.015 | -0.012 | 0.020 |
| JM-Hom | 0.302 | 0.273 | 0.032 | 0.030 | 96.4 | 0.518 | 0.188 | 0.033 | 0.032 | 94.1 | -0.039 | 25.194 | 0.029 | 0.024 | 94.4 | 0.017 | -0.012 | 0.020 |
| JM-Het | 0.311 | 3.435 | 0.034 | 0.032 | 96.1 | 0.508 | 2.162 | 0.035 | 0.035 | 93.0 | -0.039 | 25.150 | 0.029 | 0.024 | 94.3 | 0.018 | -0.013 | 0.020 |
| SMC-JM | 0.300 | 0.298 | 0.030 | 0.030 | 95.2 | 0.520 | 0.199 | 0.033 | 0.032 | 94.1 | -0.050 | 4.321 | 0.026 | 0.031 | 90.8 | 0.015 | -0.012 | 0.020 |
| Level 2 variable effect 5 times as large: | | | | | |  |  |  |  |  |  |  |  |  |  |  |  |  |
| True value | 0.301 |  |  |  |  | 0.519 |  |  |  |  | -0.130 |  |  |  |  | 0.016 | -0.012 | 0.020 |
| Full Data | 0.301 | 0.169 | 0.029 | 0.029 | 93.7 | 0.520 | 0.048 | 0.033 | 0.032 | 94.3 | -0.130 | 0.018 | 0.022 | 0.024 | 92.0 | 0.016 | -0.013 | 0.020 |
| Comp. Rec. | 0.300 | 0.376 | 0.035 | 0.036 | 92.4 | 0.517 | 0.418 | 0.039 | 0.04 | 92.8 | -0.129 | 1.022 | 0.026 | 0.031 | 88.9 | 0.015 | -0.012 | 0.020 |
| JM-Hom | 0.303 | 0.801 | 0.038 | 0.032 | 97.0 | 0.518 | 0.339 | 0.033 | 0.033 | 94.2 | -0.103 | 20.845 | 0.039 | 0.028 | 94.2 | 0.022 | -0.013 | 0.020 |
| JM-Het | 0.314 | 4.551 | 0.041 | 0.034 | 96.0 | 0.506 | 2.521 | 0.036 | 0.036 | 93.6 | -0.102 | 21.253 | 0.039 | 0.028 | 94.3 | 0.024 | -0.014 | 0.021 |
| SMC-JM | 0.302 | 0.279 | 0.032 | 0.032 | 93.9 | 0.519 | 0.023 | 0.033 | 0.033 | 93.9 | -0.130 | 0.154 | 0.026 | 0.029 | 91.6 | 0.016 | -0.013 | 0.020 |

Table f: Results of cross-level interaction scenarios, with sensitivity to larger effect than in the base-case scenario. We provide estimates for slope parameters and level 2 variance components.

| Method | $\beta_{1}$ | | | | | $\beta_{2}$ | | | | | $\beta_{3}$ | | | | | $\sigma_{u,00}$ | $\sigma_{u,01}$ | $\sigma_{u,11}$ |
| --- | --- | --- | --- | --- | --- | --- | --- | --- | --- | --- | --- | --- | --- | --- | --- | --- | --- | --- |
|  | Mean | % rel. bias | Model SE | Emp SE | % Cov | Mean | % rel. bias | Model SE | Emp SE | % Cov | Mean | % rel. bias | Model SE | Emp SE | % Cov | Mean | Mean | Mean |
| Cross-level interaction scenario: | | | | | | | | |  |  |  |  |  |  |  |  |  |  |
| True value | 0.520 |  |  |  |  | -0.035 |  |  |  |  | 0.013 |  |  |  |  | 0.016 | -0.012 | 0.020 |
| Full Data | 0.518 | 0.250 | 0.033 | 0.035 | 92.9 | -0.035 | 0.040 | 0.032 | 0.034 | 93.7 | 0.012 | 9.020 | 0.036 | 0.037 | 93.5 | 0.016 | -0.012 | 0.020 |
| Comp. Rec. | 0.517 | 0.562 | 0.040 | 0.044 | 90.6 | -0.033 | 5.185 | 0.039 | 0.041 | 92.7 | 0.011 | 18.841 | 0.044 | 0.045 | 93.1 | 0.016 | -0.012 | 0.020 |
| JM-Hom | 0.517 | 0.624 | 0.035 | 0.035 | 93.9 | -0.025 | 28.556 | 0.039 | 0.031 | 97.5 | 0.008 | 41.440 | 0.042 | 0.033 | 97.8 | 0.017 | -0.013 | 0.020 |
| JM-Het | 0.507 | 2.529 | 0.037 | 0.036 | 94.2 | -0.026 | 27.236 | 0.041 | 0.032 | 97.4 | 0.008 | 36.347 | 0.043 | 0.033 | 98.2 | 0.018 | -0.014 | 0.020 |
| SMC-JM | 0.519 | 0.203 | 0.034 | 0.036 | 92.5 | -0.032 | 8.896 | 0.037 | 0.040 | 92.2 | 0.010 | 21.744 | 0.041 | 0.043 | 92.0 | 0.015 | -0.012 | 0.019 |
| Cross-level interaction effect twice as large: | | | | | | | | |  |  |  |  |  |  |  |  |  |  |
| True value | 0.520 |  |  |  |  | -0.070 |  |  |  |  | 0.026 |  |  |  |  | 0.016 | -0.012 | 0.020 |
| Full Data | 0.521 | 0.263 | 0.033 | 0.031 | 94.7 | -0.069 | 1.591 | 0.032 | 0.034 | 92.0 | 0.026 | 2.253 | 0.036 | 0.037 | 92.9 | 0.016 | -0.012 | 0.020 |
| Comp. Rec. | 0.521 | 0.150 | 0.040 | 0.040 | 92.0 | -0.068 | 3.456 | 0.040 | 0.044 | 91.6 | 0.025 | 6.137 | 0.044 | 0.049 | 91.5 | 0.015 | -0.012 | 0.020 |
| JM-Hom | 0.519 | 0.084 | 0.035 | 0.032 | 95.1 | -0.052 | 26.614 | 0.042 | 0.033 | 95.3 | 0.018 | 30.407 | 0.043 | 0.035 | 97.5 | 0.018 | -0.013 | 0.020 |
| JM-Het | 0.510 | 1.942 | 0.038 | 0.034 | 95.5 | -0.052 | 26.349 | 0.044 | 0.033 | 95.7 | 0.018 | 29.935 | 0.044 | 0.035 | 97.7 | 0.019 | -0.014 | 0.020 |
| SMC-JM | 0.522 | 0.338 | 0.034 | 0.033 | 93.9 | -0.066 | 5.954 | 0.038 | 0.042 | 91.5 | 0.024 | 7.856 | 0.041 | 0.047 | 90.9 | 0.015 | -0.012 | 0.019 |
| Cross-level interaction effect 5 times as large: | | | | | |  |  |  |  |  |  |  |  |  |  |  |  |  |
| True value | 0.520 |  |  |  |  | -0.176 |  |  |  |  | 0.066 |  |  |  |  | 0.016 | -0.012 | 0.020 |
| Full Data | 0.519 | 0.077 | 0.033 | 0.036 | 92.5 | -0.176 | 0.103 | 0.032 | 0.035 | 92.5 | 0.064 | 1.892 | 0.036 | 0.039 | 92.4 | 0.015 | -0.012 | 0.020 |
| Comp. Rec. | 0.518 | 0.369 | 0.040 | 0.043 | 91.9 | -0.176 | 0.135 | 0.039 | 0.042 | 92.3 | 0.065 | 1.382 | 0.044 | 0.048 | 91.7 | 0.016 | -0.012 | 0.020 |
| JM-Hom | 0.518 | 0.413 | 0.037 | 0.036 | 94.3 | -0.139 | 21.051 | 0.054 | 0.036 | 94.4 | 0.050 | 23.871 | 0.045 | 0.035 | 95.4 | 0.027 | -0.017 | 0.021 |
| JM-Het | 0.506 | 2.629 | 0.039 | 0.039 | 93.2 | -0.139 | 21.229 | 0.056 | 0.037 | 95.4 | 0.051 | 22.324 | 0.046 | 0.035 | 96.0 | 0.028 | -0.018 | 0.022 |
| SMC-JM | 0.519 | 0.144 | 0.034 | 0.037 | 91.2 | -0.175 | 0.707 | 0.038 | 0.040 | 92.0 | 0.064 | 2.175 | 0.040 | 0.044 | 90.8 | 0.016 | -0.013 | 0.020 |

Table g: Results of random slope scenarios, with sensitivity to smaller clusters than in the base-case scenario. In particular, average cluster size is 5 and 15 in these two scenarios.

| Method | $\beta_{0}$ | | | | | $\beta_{1}$ | | | | | $\beta_{2}$ | | | | | $\sigma_{u,00}$ | $\sigma_{u,01}$ | $\sigma_{u,11}$ |
| --- | --- | --- | --- | --- | --- | --- | --- | --- | --- | --- | --- | --- | --- | --- | --- | --- | --- | --- |
|  | Mean | % rel. bias | Model SE | Emp SE | % Cov | Mean | % rel. bias | Model SE | Emp SE | % Cov | Mean | % rel. bias | Model SE | Emp SE | % Cov | Mean | Mean | Mean |
| Average cluster size=5 | | | | | | | | |  |  |  |  |  |  |  |  |  |  |
| True value | 0.297 |  |  |  |  | 0.511 |  |  |  |  | -0.004 |  |  |  |  | 0.015 | -0.011 | 0.021 |
| Full Data | 0.297 | 0.051 | 0.010 | 0.011 | 93.2 | 0.510 | 0.094 | 0.012 | 0.012 | 93.4 | -0.004 | 0.394 | 0.003 | 0.003 | 94.9 | 0.015 | -0.011 | 0.021 |
| Comp. Rec. | 0.288 | 2.925 | 0.011 | 0.012 | 87.8 | 0.505 | 1.128 | 0.014 | 0.014 | 92.4 | -0.004 | 1.780 | 0.004 | 0.004 | 95.8 | 0.015 | -0.011 | 0.021 |
| JM-Hom | 0.302 | 1.833 | 0.011 | 0.011 | 93.3 | 0.503 | 1.456 | 0.013 | 0.012 | 92.0 | -0.004 | 8.870 | 0.004 | 0.003 | 95.5 | 0.016 | -0.012 | 0.023 |
| JM-Het | 0.337 | 13.612 | 0.016 | 0.012 | 25.4 | 0.461 | 9.719 | 0.018 | 0.013 | 14.8 | -0.010 | 129.616 | 0.005 | 0.012 | 14.8 | 0.023 | -0.019 | 0.029 |
| SMC-JM | 0.297 | 0.039 | 0.010 | 0.011 | 93.9 | 0.51 | 0.095 | 0.012 | 0.012 | 93.4 | -0.004 | 0.547 | 0.003 | 0.003 | 95.1 | 0.015 | -0.011 | 0.021 |
| Average cluster size=15 | | | | | | | | |  |  |  |  |  |  |  |  |  |  |
| True value | 0.297 |  |  |  |  | 0.511 |  |  |  |  | -0.004 |  |  |  |  | 0.015 | -0.011 | 0.021 |
| Full Data | 0.296 | 0.213 | 0.015 | 0.015 | 95.2 | 0.512 | 0.206 | 0.017 | 0.016 | 94.5 | -0.004 | 1.428 | 0.002 | 0.003 | 94.3 | 0.015 | -0.011 | 0.021 |
| Comp. Rec. | 0.289 | 2.437 | 0.015 | 0.015 | 92.0 | 0.508 | 0.489 | 0.018 | 0.018 | 94.3 | -0.004 | 1.655 | 0.004 | 0.004 | 94.2 | 0.015 | -0.011 | 0.021 |
| JM-Hom | 0.300 | 1.197 | 0.016 | 0.015 | 96.4 | 0.506 | 0.820 | 0.018 | 0.016 | 95.2 | -0.004 | 6.672 | 0.003 | 0.003 | 95.4 | 0.016 | -0.012 | 0.022 |
| JM-Het | 0.315 | 6.267 | 0.019 | 0.015 | 90.4 | 0.488 | 4.330 | 0.021 | 0.017 | 87.8 | -0.005 | 9.939 | 0.005 | 0.004 | 98.0 | 0.019 | -0.014 | 0.025 |
| SMC-JM | 0.296 | 0.219 | 0.015 | 0.015 | 95.4 | 0.512 | 0.205 | 0.017 | 0.017 | 95.1 | -0.004 | 0.657 | 0.003 | 0.003 | 93.6 | 0.015 | -0.011 | 0.021 |

Table h: Results of base case random slope scenarios, with sensitivity to fewer clusters (6,12) and to presence of 20% systematically missing data. We provide estimates for all parameters and level 2 variance components.

| Method | $\beta_{0}$ | | | | | $\beta_{1}$ | | | | | $\beta_{2}$ | | | | | $\sigma_{u,00}$ | $\sigma_{u,01}$ | $\sigma_{u,11}$ |
| --- | --- | --- | --- | --- | --- | --- | --- | --- | --- | --- | --- | --- | --- | --- | --- | --- | --- | --- |
|  | Mean | % rel. bias | Model SE | Emp SE | % Cov | Mean | % rel. bias | Model SE | Emp SE | % Cov | Mean | % rel. bias | Model SE | Emp SE | % Cov | Mean | Mean | Mean |
| 6 clusters only: | | | | | | | | |  |  |  |  |  |  |  |  |  |  |
| True value | 0.297 |  |  |  |  | 0.511 |  |  |  |  | -0.004 |  |  |  |  | 0.015 | -0.011 | 0.021 |
| Full Data | 0.297 | 0.145 | 0.049 | 0.051 | 89.1 | 0.508 | 0.431 | 0.058 | 0.061 | 90.3 | -0.004 | 0.670 | 0.002 | 0.002 | 95.1 | 0.015 | -0.011 | 0.022 |
| Comp. Rec. | 0.292 | 1.502 | 0.050 | 0.052 | 88.5 | 0.505 | 1.001 | 0.058 | 0.062 | 89.8 | -0.004 | 0.017 | 0.003 | 0.003 | 94.0 | 0.015 | -0.012 | 0.022 |
| JM-Hom | 0.297 | 0.288 | 0.050 | 0.052 | 89.0 | 0.508 | 0.535 | 0.058 | 0.062 | 90.3 | -0.004 | 8.981 | 0.003 | 0.003 | 95.2 | 0.016 | -0.012 | 0.022 |
| JM-Het | 0.305 | 2.965 | 0.051 | 0.055 | 88.8 | 0.499 | 2.223 | 0.058 | 0.064 | 89.5 | -0.004 | 6.120 | 0.004 | 0.003 | 97.7 | 0.016 | -0.012 | 0.021 |
| SMC-JM | 0.297 | 0.169 | 0.049 | 0.052 | 88.8 | 0.508 | 0.450 | 0.058 | 0.062 | 90.3 | -0.004 | 0.289 | 0.003 | 0.003 | 93.9 | 0.016 | -0.012 | 0.022 |
| 12 clusters only: | | | | | | | | |  |  |  |  |  |  |  |  |  |  |
| True value | 0.297 |  |  |  |  | 0.511 |  |  |  |  | -0.004 |  |  |  |  | 0.015 | -0.011 | 0.021 |
| Full Data | 0.296 | 0.228 | 0.035 | 0.036 | 91.1 | 0.512 | 0.321 | 0.042 | 0.043 | 92.3 | -0.004 | 1.624 | 0.002 | 0.002 | 94.7 | 0.015 | -0.011 | 0.022 |
| Comp. Rec. | 0.292 | 1.708 | 0.035 | 0.036 | 90.5 | 0.508 | 0.411 | 0.042 | 0.043 | 91.6 | -0.004 | 1.715 | 0.003 | 0.003 | 94.2 | 0.015 | -0.011 | 0.021 |
| JM-Hom | 0.297 | 0.004 | 0.035 | 0.036 | 91.4 | 0.512 | 0.218 | 0.042 | 0.043 | 92.4 | -0.004 | 9.284 | 0.003 | 0.003 | 96.5 | 0.015 | -0.011 | 0.021 |
| JM-Het | 0.300 | 1.235 | 0.035 | 0.037 | 91.7 | 0.507 | 0.655 | 0.042 | 0.043 | 92.4 | -0.004 | 0.065 | 0.004 | 0.003 | 97.8 | 0.015 | -0.011 | 0.021 |
| SMC-JM | 0.296 | 0.226 | 0.035 | 0.036 | 91.0 | 0.512 | 0.322 | 0.042 | 0.043 | 92.4 | -0.004 | 1.827 | 0.003 | 0.003 | 95.2 | 0.015 | -0.011 | 0.022 |
| Systematically missing data: | | | | | |  |  |  |  |  |  |  |  |  |  |  |  |  |
| True value | 0.297 |  |  |  |  | 0.511 |  |  |  |  | -0.004 |  |  |  |  | 0.015 | -0.011 | 0.021 |
| Full Data | 0.296 | 0.163 | 0.029 | 0.029 | 92.2 | 0.509 | 0.314 | 0.034 | 0.034 | 94.2 | -0.004 | 1.686 | 0.002 | 0.002 | 94.9 | 0.015 | -0.011 | 0.021 |
| Comp. Rec. | 0.291 | 1.981 | 0.029 | 0.030 | 92.1 | 0.507 | 0.783 | 0.034 | 0.034 | 94.3 | -0.004 | 3.164 | 0.003 | 0.003 | 95.8 | 0.015 | -0.011 | 0.021 |
| JM-Hom | 0.298 | 0.454 | 0.029 | 0.029 | 92.4 | 0.507 | 0.715 | 0.034 | 0.034 | 94.5 | -0.004 | 6.877 | 0.003 | 0.003 | 97.4 | 0.016 | -0.011 | 0.021 |
| JM-Het | 0.308 | 3.695 | 0.032 | 0.032 | 92.9 | 0.496 | 2.876 | 0.036 | 0.036 | 93.3 | -0.004 | 4.763 | 0.004 | 0.003 | 98.6 | 0.017 | -0.012 | 0.022 |
| SMC-JM | 0.296 | 0.166 | 0.029 | 0.029 | 92.3 | 0.509 | 0.303 | 0.034 | 0.034 | 94.0 | -0.004 | 2.808 | 0.003 | 0.003 | 95.1 | 0.015 | -0.011 | 0.021 |

Table i: Results of scenarios with different substantive model. In particular, here we investigate GLMER (binomial logit) and CLMM.

| Method | $\beta_{0}$ | | | | | $\beta_{1}$ | | | | | $\beta_{2}$ | | | | | $\beta_{0\vert1}$ | $\beta_{1\vert2}$ | $\beta_{2\vert3}$ |
| --- | --- | --- | --- | --- | --- | --- | --- | --- | --- | --- | --- | --- | --- | --- | --- | --- | --- | --- |
|  | Mean | % rel. bias | Model SE | Emp SE | % Cov | Mean | % rel. bias | Model SE | Emp SE | % Cov | Mean | % rel. bias | Model SE | Emp SE | % Cov | Mean | Mean | Mean |
| GLMER substantive model: | | | | | | | | |  |  |  |  |  |  |  |  |  |  |
| True value | 2.678 |  |  |  |  | -0.081 |  |  |  |  | -0.216 |  |  |  |  |  |  |  |
| Full Data | 2.709 | 1.161 | 0.697 | 0.841 | 92.8 | -0.075 | 7.536 | 0.179 | 0.183 | 94.0 | -0.220 | 1.716 | 0.090 | 0.091 | 94.0 |  |  |  |
| Comp.Rec. | 2.019 | 24.596 | 0.725 | 0.883 | 73.4 | -0.074 | 8.681 | 0.235 | 0.250 | 93.0 | -0.220 | 2.075 | 0.118 | 0.125 | 93.0 |  |  |  |
| JM-Hom | 2.714 | 1.348 | 0.702 | 0.849 | 93.0 | -0.080 | 1.449 | 0.209 | 0.215 | 95.2 | -0.219 | 1.290 | 0.104 | 0.107 | 94.6 |  |  |  |
| JM-Het | 2.715 | 1.395 | 0.708 | 0.852 | 93.3 | -0.077 | 4.351 | 0.247 | 0.233 | 96.0 | -0.222 | 2.774 | 0.130 | 0.116 | 95.6 |  |  |  |
| SMC-JM | 2.711 | 1.248 | 0.703 | 0.848 | 93.1 | -0.075 | 6.487 | 0.209 | 0.215 | 94.7 | -0.219 | 1.369 | 0.104 | 0.106 | 94.4 |  |  |  |
| CLMM substantive model: | | | | | | | | |  |  |  |  |  |  |  |  |  |  |
| True value |  |  |  |  |  | -0.035 |  |  |  |  | -0.110 |  |  |  |  | -1.902 | -0.473 | 2.268 |
| Full Data |  |  |  |  |  | -0.075 | 6.487 | 0.209 | 0.215 | 94.7 | -0.219 | 1.369 | 0.104 | 0.106 | 94.4 | -1.886 | -0.456 | 2.289 |
| Comp.Rec. |  |  |  |  |  | -0.035 |  |  |  |  | -0.110 |  |  |  |  | -1.691 | -0.273 | 2.480 |
| JM-Hom |  |  |  |  |  | -0.034 | 2.348 | 0.100 | 0.098 | 95.6 | -0.110 | 0.397 | 0.050 | 0.050 | 94. 3 | -1.886 | -0.456 | 2.290 |
| JM-Het |  |  |  |  |  | -0.034 | 2.243 | 0.121 | 0.123 | 93.8 | -0.110 | 0.267 | 0.060 | 0.060 | 95.4 | -1.887 | -0.457 | 2.290 |
| SMC-JM |  |  |  |  |  | -0.034 | 3.591 | 0.111 | 0.110 | 95.6 | -0.108 | 2.107 | 0.055 | 0.055 | 95.5 | -1.884 | -0.453 | 2.293 |

Table l: Results of random slope scenarios, adding auxiliary variables. In the first scenario, this is related to PAR use. In the second, to the outcome.

| Method | $\beta_{0}$ | | | | | $\beta_{1}$ | | | | | $\beta_{2}$ | | | | | $\sigma_{u,00}$ | $\sigma_{u,01}$ | $\sigma_{u,11}$ |
| --- | --- | --- | --- | --- | --- | --- | --- | --- | --- | --- | --- | --- | --- | --- | --- | --- | --- | --- |
|  | Mean | % rel. bias | Model SE | Emp SE | % Cov | Mean | % rel. bias | Model SE | Emp SE | % Cov | Mean | % rel. bias | Model SE | Emp SE | % Cov | Mean | Mean | Mean |
| Auxiliary variable related to covariate: | | | | | | | | |  |  |  |  |  |  |  |  |  |  |
| True value | 0.297 |  |  |  |  | 0.511 |  |  |  |  | -0.004 |  |  |  |  | 0.015 | -0.011 | 0.021 |
| Full Data | 0.295 | 0.464 | 0.029 | 0.028 | 95.1 | 0.512 | 0.305 | 0.034 | 0.034 | 93.5 | -0.004 | 0.394 | 0.002 | 0.002 | 94.5 | 0.015 | -0.011 | 0.021 |
| Comp. Rec. | 0.290 | 2.232 | 0.029 | 0.028 | 93.8 | 0.509 | 0.247 | 0.034 | 0.034 | 93.5 | -0.004 | 0.820 | 0.003 | 0.003 | 94.4 | 0.015 | -0.011 | 0.021 |
| JM-Hom | 0.360 | 21.335 | 0.037 | 0.032 | 60.4 | 0.439 | 14.066 | 0.038 | 0.035 | 53.6 | -0.007 | 54.802 | 0.004 | 0.008 | 53.6 | 0.021 | -0.015 | 0.023 |
| JM-Het | 0.322 | 8.655 | 0.034 | 0.035 | 89.6 | 0.481 | 5.697 | 0.038 | 0.041 | 85.5 | -0.005 | 18.270 | 0.004 | 0.006 | 85.6 | 0.018 | -0.013 | 0.022 |
| SMC-JM | 0.295 | 0.508 | 0.029 | 0.028 | 95.3 | 0.512 | 0.327 | 0.034 | 0.034 | 93.8 | -0.004 | 0.468 | 0.002 | 0.003 | 94.1 | 0.015 | -0.011 | 0.021 |
| Auxiliary variable related to the outcome: | | | | | | | | |  |  |  |  |  |  |  |  |  |  |
| True value | 0.297 |  |  |  |  | 0.511 |  |  |  |  | -0.004 |  |  |  |  | 0.015 | -0.011 | 0.021 |
| Full Data | 0.296 | 0.066 | 0.029 | 0.031 | 92.2 | 0.511 | 0.049 | 0.034 | 0.035 | 93.1 | -0.004 | 0.943 | 0.002 | 0.002 | 96.7 | 0.016 | -0.011 | 0.021 |
| Comp. Rec. | 0.291 | 1.738 | 0.029 | 0.031 | 91.8 | 0.508 | 0.573 | 0.034 | 0.035 | 92.9 | -0.004 | 3.507 | 0.003 | 0.003 | 94.9 | 0.015 | -0.011 | 0.021 |
| JM-Hom | 0.313 | 5.405 | 0.033 | 0.034 | 91.3 | 0.492 | 3.675 | 0.038 | 0.038 | 92.2 | -0.004 | 6.452 | 0.003 | 0.003 | 97.3 | 0.017 | -0.012 | 0.022 |
| JM-Het | 0.313 | 5.611 | 0.033 | 0.034 | 90.9 | 0.491 | 3.752 | 0.037 | 0.037 | 90.6 | -0.005 | 7.555 | 0.005 | 0.004 | 97.2 | 0.017 | -0.013 | 0.022 |
| SMC-JM | 0.297 | 0.300 | 0.029 | 0.031 | 91.9 | 0.510 | 0.172 | 0.034 | 0.035 | 93.2 | -0.005 | 10.961 | 0.003 | 0.003 | 93.6 | 0.016 | -0.011 | 0.022 |

Table m: Results of random slope scenarios, generating data from different mechanisms as a sensitivity analysis. .

| Method | $\beta_{0}$ | | | | | $\beta_{1}$ | | | | | $\beta_{2}$ | | | | | $\sigma_{u,00}$ | $\sigma_{u,01}$ | $\sigma_{u,11}$ |
| --- | --- | --- | --- | --- | --- | --- | --- | --- | --- | --- | --- | --- | --- | --- | --- | --- | --- | --- |
|  | Mean | % rel. bias | Model SE | Emp SE | % Cov | Mean | % rel. bias | Model SE | Emp SE | % Cov | Mean | % rel. bias | Model SE | Emp SE | % Cov | Mean | Mean | Mean |
| Sensitivity 1: | | | | | | | | |  |  |  |  |  |  |  |  |  |  |
| True value | 0.297 |  |  |  |  | 0.511 |  |  |  |  | -0.004 |  |  |  |  | 0.015 | -0.011 | 0.021 |
| Full Data | 0.297 | 0.071 | 0.027 | 0.027 | 94.9 | 0.511 | 0.004 | 0.033 | 0.033 | 92.9 | -0.004 | 1.178 | 0.005 | 0.005 | 94.1 | 0.015 | -0.011 | 0.021 |
| Comp. Rec. | 0.292 | 1.417 | 0.027 | 0.027 | 94.1 | 0.506 | 0.801 | 0.033 | 0.033 | 92.7 | -0.004 | 5.143 | 0.006 | 0.006 | 94.4 | 0.015 | -0.011 | 0.021 |
| JM-Hom | 0.300 | 1.207 | 0.028 | 0.027 | 95.1 | 0.505 | 1.162 | 0.033 | 0.032 | 93.3 | -0.011 | 151.731 | 0.010 | 0.023 | 74.9 | 0.015 | -0.011 | 0.021 |
| JM-Het | 0.300 | 1.003 | 0.028 | 0.027 | 95.2 | 0.505 | 1.041 | 0.033 | 0.033 | 92.7 | 0.000 | 96.139 | 0.008 | 0.006 | 96.2 | 0.015 | -0.011 | 0.021 |
| SMC-JM | 0.297 | 0.068 | 0.027 | 0.027 | 94.8 | 0.511 | 0.003 | 0.033 | 0.033 | 92.7 | -0.004 | 5.372 | 0.006 | 0.006 | 94.3 | 0.015 | -0.011 | 0.021 |
| Sensitivity 2, with quadratic relationship between covariates: | | | | | | | | |  |  |  |  |  |  |  |  |  |  |
| True value | 0.297 |  |  |  |  | 0.511 |  |  |  |  | -0.004 |  |  |  |  | 0.015 | -0.011 | 0.021 |
| Full Data | 0.295 | 0.496 | 0.027 | 0.027 | 93.9 | 0.512 | 0.321 | 0.032 | 0.033 | 93.1 | -0.004 | 4.817 | 0.005 | 0.005 | 94.3 | 0.015 | -0.011 | 0.021 |
| Comp. Rec. | 0.291 | 1.998 | 0.027 | 0.027 | 92.3 | 0.508 | 0.421 | 0.033 | 0.033 | 92.9 | -0.005 | 7.186 | 0.006 | 0.006 | 95.3 | 0.015 | -0.011 | 0.021 |
| JM-Hom | 0.301 | 1.403 | 0.028 | 0.027 | 94.3 | 0.505 | 0.992 | 0.033 | 0.032 | 94.4 | -0.011 | 165.289 | 0.012 | 0.026 | 74.3 | 0.015 | -0.011 | 0.021 |
| JM-Het | 0.298 | 0.444 | 0.028 | 0.027 | 94.2 | 0.507 | 0.678 | 0.033 | 0.033 | 93.1 | -0.001 | 82.356 | 0.008 | 0.007 | 97.3 | 0.015 | -0.011 | 0.021 |
| SMC-JM | 0.295 | 0.504 | 0.027 | 0.027 | 93.7 | 0.512 | 0.318 | 0.032 | 0.033 | 93.0 | -0.004 | 3.842 | 0.006 | 0.006 | 95.1 | 0.015 | -0.011 | 0.021 |

**Code to impute missing data using SMC-JM as implemented in the R package jomo.**

**Section 4.1:**

#Define the substantive analysis model:
analysis.model<-as.formula(Y~par+scaled.age+(1|clus))

# Impute data in data.miss n.imp times, using n.burn and n.between burn-in and between-imp iterations:
imps<-jomo.lmer(data.miss, formula = analysis.model, nburn=n.burn, nbetween=n.between, nimp=n.imp)

# Convert to mitml list format:

implist <- jomo2mitml.list(imps)

# Fit substantive model on each imputation:

fit.m<-with(implist, lmer(Y~par+scaled.age+(1|clus)))

# Pool results, estimating variance components as well:

fit.JM.SMC<-testEstimates(fit.m, var.comp=T)

**Section 4.2:**

#Define the substantive analysis model:
analysis.model<-as.formula(Y~par+scaled.age+(1+par|clus))

# Impute data in data.miss n.imp times:
imps<-jomo.lmer(data.miss, formula = analysis.model, nburn=n.burn, nbetween=n.between, nimp=n.imp)

**Section 4.3:**

# Substantive analysis model with quadratic effect:
analysis.model<-as.formula(Y~par+scaled.age+ I(scaled.age^2)+(1+par|clus))

# Substantive analysis model with cubic effect:
analysis.model<-as.formula(Y~par+scaled.age+ I(scaled.age^3)+(1+par|clus))

# Substantive analysis model with interaction:
analysis.model<-as.formula(Y~par*scaled.age+(1+par|clus))

**Section 4.4:**

#Define the substantive analysis model:
analysis.model<-as.formula(Y~par+scaled.age+(1+par|clus))

# Define level for each variable in the dataset:

data.miss<-data.sim[,c("Y","par","scaled.age", “clus”)]

mylevel<-c(1,1,2,2)

# Impute data in data.miss n.imp times:
imps<-jomo.lmer(data.miss, formula = analysis.model, level=mylevel, nimp=n.imp)

**Section 4.5:**

#Define the substantive analysis model:
analysis.model<-as.formula(Y~par+scaled.age+(1+par|clus))

# Define variables to include in imputation model:

data.miss<-data.sim[,c("Y","par","scaled.age", “Xaux”,“clus”)]

# Impute data in data.miss n.imp times:
imps<-jomo.lmer(data.miss, formula = analysis.model)

Section 4.6:

#Define the substantive analysis model:
analysis.model<-as.formula(Y~par+scaled.age+(1|clus))

# Impute when Y is binary:
imps<-jomo.glmer(data.miss, formula = analysis.model)

# Impute when Y is ordinal:
imps<-jomo.clmm(data.miss, formula = analysis.model)
